# The Dutch chronic lower limb-threatening ischemia registry (THRILLER): A study protocol for popliteal and infrapopliteal endovascular interventions

**DOI:** 10.1371/journal.pone.0288912

**Published:** 2023-07-20

**Authors:** Michael J. Nugteren, Constantijn E. V. B. Hazenberg, George P. Akkersdijk, Olaf J. Bakker, Maarten K. Dinkelman, Bram Fioole, Daniel A. F. van den Heuvel, Jan M. M. Heyligers, Jan-Willem Hinnen, Maurice Pierie, Olaf Schouten, Michiel A. Schreve, Bart A. N. Verhoeven, Gert J. de Borst, Çağdaş Ünlü

**Affiliations:** 1 Department of Vascular Surgery, Noordwest Ziekenhuisgroep, Alkmaar, The Netherlands; 2 Department of Vascular Surgery, University Medical Center Utrecht, Utrecht, The Netherlands; 3 Department of Vascular Surgery, Maasstad Ziekenhuis, Rotterdam, The Netherlands; 4 Department of Vascular Surgery, St. Antonius Ziekenhuis, Nieuwegein, The Netherlands; 5 Department of Vascular Surgery, Elisabeth-TweeSteden Ziekenhuis, Tilburg, The Netherlands; 6 Department of Radiology, St. Antonius Ziekenhuis, Nieuwegein, The Netherlands; 7 Department of Vascular Surgery, Jeroen Bosch Ziekenhuis, ‘s-Hertogenbosch, The Netherlands; 8 Department of Vascular Surgery, Isala Ziekenhuis, Zwolle, The Netherlands; Ataturk University Faculty of Medicine, TURKEY

## Abstract

**Introduction:**

Chronic limb-threatening ischemia (CLTI) is the end stage of peripheral arterial disease (PAD) and is associated with high amputation rates, mortality and disease-related health care costs. In infrapopliteal arterial disease (IPAD), endovascular revascularization should be considered for the majority of anatomical and clinical subgroups of CLTI. However, a gap of high-quality evidence exists in this field. The aim of the Dutch Chronic Lower Limb-Threatening Ischemia Registry (THRILLER) is to collect real world data on popliteal and infrapopliteal endovascular interventions.

**Methods:**

THRILLER is a clinician-driven, prospective, multicenter, observational registry including all consecutive patients that undergo a popliteal or infrapopliteal endovascular intervention in seven Dutch hospitals. We estimate that THRILLER will include 400–500 interventions annually. Standardized follow-up visits with wound monitoring, toe pressure measurement and duplex ultrasonography will be scheduled at 6–8 weeks and 12 months after the intervention. The independent primary endpoints are primary patency, limb salvage and amputation free survival. Patients must give informed consent before participation and will be included according to predefined reporting standards. A data log of patients who meet the inclusion criteria but are not included in the registry will be maintained. We intend to conduct the first interim analysis two years after the start of inclusion. The results will be published in a scientific journal.

**Discussion:**

Despite innovations in medical therapy and revascularization techniques, patients with CLTI undergoing endovascular revascularization still have a moderate prognosis. Previous prospective cohort studies were hampered by small sample sizes or heterogeneous reporting. Randomized controlled trials (RCTs) have high costs, potential conflicts of interest and give a limited reflection of daily practice. THRILLER aims to provide the largest prospective well phenotyped up-to-date dataset on treatment outcomes in CLTI patients to answer multiple underexplored research questions regarding diagnostics, medication, patient selection, treatment strategies and post intervention follow-up.

## Introduction

Peripheral artery disease (PAD) affects over 200 million people worldwide and the prevalence is expected to increase rapidly in the next decades [1]. About 5–10% of patients with PAD will progress to the end stage of PAD, also known as chronic limb-threatening ischemia (CLTI) which is characterized by ischemic rest pain and tissue loss (ulceration or gangrene) [2]. CLTI is associated with high amputation and mortality rates [3]. Moreover, the disease-related health care costs of CLTI patients in the Netherlands are estimated to be €7,000 to €10,000 per year, compared to €2,000 per year for patients with intermittent claudication [4].

The global prevalence of diabetes mellitus (DM) is currently about 463 million people, but is expected to grow to 700 million people by 2045 [5]. Because DM is the main cause of CLTI and infrapopliteal arterial disease (IPAD), the burden of the latter two is expected to grow proportionally.

The optimal revascularization approach for patients with CLTI should be based on an individualized assessment of patient risk, limb severity and anatomic complexity. Although IPAD is hard to treat due to small vessel diameter, long lesion length, multilevel disease and severe calcification [6], endovascular revascularization should be offered or at least considered for most anatomic and clinical subgroups of CLTI [7]. Endovascular revascularization offers the advantages of minimally invasiveness, local anesthesia, shorter hospital stays, potentially reduced costs and favorable short-term outcomes, but is associated with more reinterventions and recurrence of CLTI [8, 9]. In patients eligible for both open and endovascular revascularization, the BEST-CLI trial found a lower rate of major adverse limb events (MALE) or death in the bypass group, which was mainly driven by a lower rate of major reinterventions [10]. In contrast, in the BASIL-2 trial, endovascular revascularization was associated with a better amputation-free survival (AFS), which was largely driven by fewer deaths [11].

The most recent global vascular guidelines (GVG) on CLTI state that plain old balloon angioplasty (POBA) remains the primary endovascular approach for anatomically suitable IPAD, as current evidence is inadequate to support other, more complex or innovative techniques [7]. Previous meta-analyses on POBA in IPAD reported a 60% primary patency, 15% limb salvage and 14% mortality at 1 year [12–14]. These rates did not change over the last few decades, despite innovations in medical therapy and revascularization techniques [15, 16], which indicates substantial room for improvement in this field. Large-scale, multicenter, prospective studies with inclusion criteria that encompass the real-world patient are warranted to generate relevant and generalizable data that can be used to develop evidence-based standards to treat this complex disease [7].

The Du**T**c**H** ch**R**on**I**c **L**ower **L**imb-threatening isch**E**mia **R**egistry (THRILLER) is designed to collect real world data on patients with CLTI undergoing popliteal and infrapopliteal endovascular interventions to answer multiple underexplored research questions.

## Materials and methods

### Study design

THRILLER is a clinician-driven, prospective, national, multicenter, observational registry including all consecutive patients that undergo a popliteal or infrapopliteal endovascular intervention for CLTI in seven Dutch hospitals (NTR ID: NL 9192; URL: https://trialregister.nl/trial/9192). All consecutive patients matching the inclusion criteria and being treated as of February 2021 are included. THRILLER is an ongoing registry in which as many patients as possible will be included according to predefined reporting standards. Hence, a well phenotyped CLTI cohort is created including both procedural data as well as mid-term follow-up outcomes to answer multiple underexplored research questions (Table 1).

**Table 1:** Study objectives of THRILLER.

Main objective 1	To provide real world data about patients undergoing (infra)popliteal endovascular interventions for chronic limb-threatening ischemia (CLTI).
Main objective 2	To determine the prognostic value of various baseline and lesion characteristics on outcomes after an (infra)popliteal endovascular intervention.
Main objective 3	To determine which subgroups of CLTI patients benefit the most from (infra)popliteal endovascular revascularization and which subgroups are more likely to benefit from an alternative treatment.
Objective 4	To investigate the additional value of duplex ultrasonography (DUS) surveillance after (infra)popliteal interventions for CLTI.
Objective 5	To assess the predictive value of (change in) toe pressure acceleration time measured by photoplethysmography on outcomes after endovascular interventions for CLTI.
Objective 6	To assess the role and additional value of specific endovascular devices in the (infra)popliteal arteries.
Objective 7	To assess the efficacy and safety of different antithrombotic strategies after (infra)popliteal endovascular interventions.
Objective 8	To assess the safety and feasibility of endovascular revascularization in various anatomical lesions in which the efficacy is considered questionable, e.g. below the ankle lesions or long multilevel occlusions.

### Inclusion and exclusion criteria

To participate, patients must be at least 18 years old and diagnosed with symptomatic PAD. Patients are primarily included based on the treated anatomical location. Infrapopliteal lesions are often a continuum from the popliteal artery, which is why we decided to include interventions in this artery as well (from P1 to P3). This study is based on an intention-to-treat (ITT) basis. Patients who undergo endovascular interventions in the specified anatomical locations without technical and/or procedural success are also included, as are patients who undergo interventions with simultaneous proximal endovascular procedures in the superficial femoral or inflow arteries, or hybrid procedures. Patients must give informed consent before participation.

Patients undergoing interventions for acute limb ischemia (ALI), distal embolization after a proximal intervention, aneurysmal disease and nonatherosclerotic arterial disease are excluded. Patients who are unable to give informed consent due to a language barrier or lack of comprehension are also excluded.

### Data collection

Data will be collected from electronic health records by local and coordinating investigators and recorded in a web-based case report form (eCRF; Castor EDC, New York, USA). Data regarding patient demographics, comorbidities, medication, medical examinations and imaging, lesion characteristics, procedural characteristics, follow-up and outcomes will be collected in accordance with the international consortium of vascular registries consensus recommendations for peripheral revascularization (S1 File) [17]. Also, a data log will be maintained of patients who meet the inclusion criteria but are not included in the registry. The data in the eCRF will be checked every four months for consistency and completeness. At the time of publication, all data underlying the findings will be made fully available without restriction.

### Endpoint definitions

The independent primary endpoints are primary patency, limb salvage and AFS. Primary patency is defined as freedom from a restenosis > 50%, as measured on magnetic resonance angiography (MRA), computed tomography angiography (CTA), digital subtraction angiography (DSA) or duplex ultrasonography (DUS) with a peak systolic velocity-ratio of > 2.4. Limb salvage is defined as freedom from major amputation (above the ankle). AFS is defined as survival free from major amputation. Secondary endpoints are further listed in Table 2.

**Table 2:** All endpoints of THRILLER.

Study endpoints	Description/definition
***Primary endpoints***	
Primary patency	Freedom from a restenosis > 50%, as measured on MRA, CTA, DSA or DUS (PSV-ratio > 2.4).
Limb salvage	Freedom from major amputation (above the ankle).
Amputation free survival (AFS)	Survival free from major amputation.
***Secondary endpoints***	
Technical success	A residual diameter stenosis ≤ 50% for angioplasty and < 30% for stenting by angiography at the end of the procedure.
Procedural success	Both acute technical success and absence of major adverse events (death, stroke, myocardial infarction, acute thrombosis or onset of limb ischemia, and/or need for emergent vascular surgery) within 72 hours of the index procedure.
Procedural complications	Acute thrombosis, distal embolization, perforation or device failure.
Wound healing and recurrent CLTI symptoms	Defined with the Fontaine and Rutherford classifications. Also time to wound healing and any recurrence of CLTI symptoms is recorded.
Minor amputations	Amputations below the ankle.
Limb-based patency	Maintained patency of the target arterial path, i.e. the selected continuous route of in-line flow from groin to ankle.
Primary assisted patency	The durability of an intervention that failed initially but not to the level of thrombosis and was retreated.
Secondary patency	The durability of a second intervention after the initial intervention failed to the level of thrombosis and was retreated.
Clinically driven target lesion revascularization (CD-TLR)	Any repeat endovascular intervention or surgical bypass grafting resulting from a significant restenosis at the level of the treated lesion in the presence of clinical deterioration.
Major adverse cardiac events (MACE)	A composite of myocardial infarction, ischemic stroke and death due to a cardiovascular cause.
Major adverse limb events (MALE)	A composite of all target limb reinterventions and major amputations.
Reintervention and AFS (RAFS)	A composite of all target limb reinterventions and AFS.
TIMI major bleeding	A composite of fatal bleeding, intracranial bleeding and bleeding with a hemoglobin decrease of ≥ 5 g/dL (3.1 mmol/L).
Overall survival	Freedom from death of any cause.

CTA = computed tomography angiography. DSA = digital subtraction angiography. DUS = duplex ultrasonography. MRA = magnetic resonance angiography. PSV = peak systolic velocity.

In the case of ambiguity or controversy an endpoint will be assessed by an independent clinical adjudication committee consisting of at least three vascular specialists (M.N., C.H. Ç.Ü.).

### Study protocol

The use of optimal medical therapy (OMT) is not mandatory to be included in the study. However, there is a recommendation to follow the guidelines regarding OMT, such as the use of an antiplatelet agent, statin therapy and control of hypertension and DM in all patients with CLTI. Antithrombotic use is documented pre-operatively, postoperatively and 6–8 weeks, 6 months, 12 months and 24 months after the intervention. Furthermore, all patients are scored for patient risk, limb severity (WIfI score) and anatomic complexity (GLASS score) according to the GVG proposed approach on CLTI [7].

**Fig 1:**
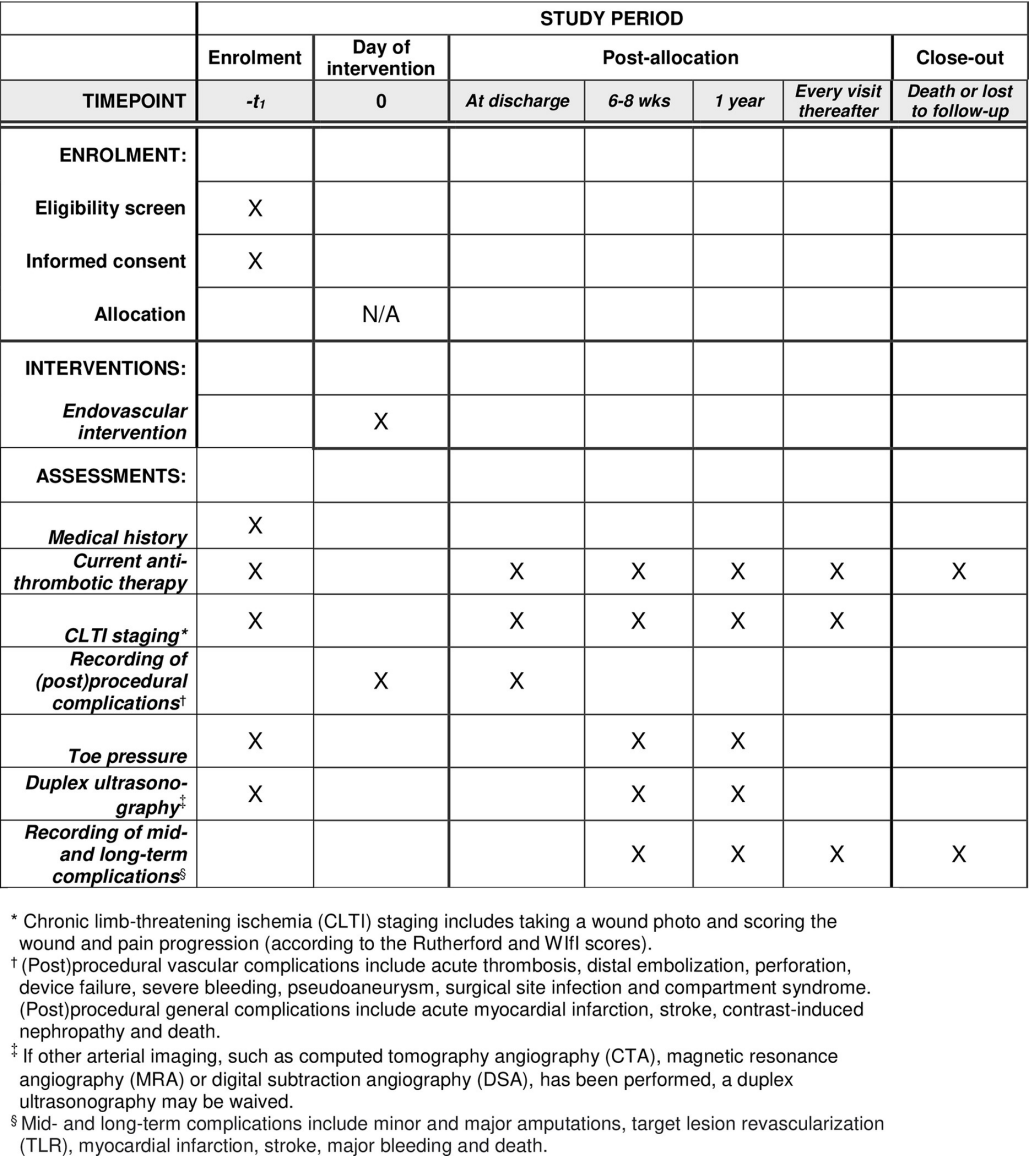
The schedule of enrolment, interventions, and assessments in the Dutch chronic lower limb-threating ischemia registry (THRILLER).

All patients in the registry will visit the outpatient clinic approximately 6–8 weeks and 12 months after the index procedure. At each of these follow-up visits, wound progression is scored (according to the Rutherford and WIfI scores) [7], wound photos are taken and patients are assessed for adverse events. Additionally, a systolic toe pressure measurement and DUS of the treated lesions are performed to check patency (Fig 1). The remaining follow-up visits and examinations are scheduled at the discretion of the treating physician. However, the results of these follow-up visits, in terms of wound healing and adverse events, will be documented in the database. Although there will be no mandatory visits after 12 months, the follow-up period will only end when patients are lost from follow-up or decease.

### Sample size

Due to the presence of multiple research questions and the ongoing character of this registry, the planned sample size could not be calculated statistically. However, we estimate that 60–70 interventions per participating center will be included annually, leading to a total inclusion rate of 400–500 interventions per year.

### Patient and public involvement

We believe that patient and public involvement is a relevant issue when designing new studies. However, due to the feasibility nature of this registry, we chose at this stage not to involve patients in the design of the study. In a future phase, we will seek patient input to determine the study design of the ongoing nationwide registry, e.g. to specify research questions and study outcomes.

### Statistical analysis

Continuous variables will be presented as mean ± standard deviation and categorical variables as absolute number and proportion of the study population. Baseline characteristics will be compared using the chi-squared test for categorical variables and the two-sample t-test for continuous variables. All outcomes will be estimated with Kaplan-Meier survival curves, and if applicable, differences between groups will be compared with the log-rank test, e.g. between different antithrombotic strategies. Findings with a P-value < 0.05 will be considered statistically significant.

### Ethics and dissemination

This study is conducted in accordance with the ethical principles of the Declaration of Helsinki and good clinical practice guidelines. The study protocol and informed consent have been reviewed and approved by the Amsterdam medical ethics committee, as well as the local ethical committees of each participating center. All patients will have given informed consent before inclusion in the registry. In six centers patients must give written consent, while in one center the local ethical committee agreed to verbal consent and a clear accompanying notation in the electronic health record.

We intend to conduct the first interim analysis two years after the start of inclusion, of which the results will be published in a scientific journal. The interim analysis will focus on procedural and short-term outcomes. Based on the results of this analysis, the research protocol will be adjusted if necessary.

## Discussion and conclusion

A lack of high-quality evidence exists for the diagnosis, treatment and follow-up of patients with CLTI and IPAD. Regarding the most recent guidelines on the management of CLTI, more than half of all recommendations were based on expert consensus or low quality evidence [7]. These guidelines also state that currently few countries maintain national registries for CLTI and therefore address the importance of ongoing surveillance with the use of well-designed, large, prospective, observational studies [7].

Prospective studies can be broadly classified into randomized controlled trials (RCTs) and registry-based studies. RCTs have the best methodological design and are less prone to selection bias than registries because patients are randomly assigned to a certain treatment. However, RCTs have a number of limitations, including high costs, long completion times, potential conflicts of interest and most importantly, the complexity regarding inclusion criteria [7]. Most RCTs apply strict inclusion criteria, which sometimes results in up to 50% of the total target population not meeting the inclusion criteria [18]. CLTI and IPAD are very heterogeneous in terms of clinical presentation, comorbidities and anatomical characteristics, such as multilevel disease, lesion morphology and grade of calcification. The available RCTs are scarce and results are not widely generalizable due to multiple exclusion criteria. Registry-based studies provide better real-world data and may better reflect daily treatment practice than RCTs.

The Society for Vascular Surgery (SVS) Vascular Quality Initiative (VQI) maintains 14 registries with demographic, clinical, procedural and outcomes data from more than a million vascular procedures [19]. VQI registries on infra-inguinal bypass and peripheral vascular interventions included thousands of patients with CLTI to date. However, the main limitation of this registry is the voluntary, self-reporting nature of this database leading to a high risk of selection bias. Also, in a study on CLTI patients using VQI data, only 1319 of the 5252 infrapopliteal endovascular interventions had sufficient follow-up data (> 9 months) to be included in the final analysis [20]. The excluded patients featured worse baseline characteristics than the patients who had enough follow-up. These two limitations raise serious concerns about the generalizability of the outcomes of VQI data. Lastly, VQI registries lack a standardized follow-up with imaging and several important variables, e.g. regarding anatomical complexity, are not documented [20]. THRILER addresses these issues through its design to include all consecutive eligible patients and by maintaining a data log of patients who meet the inclusion criteria but are eventually not included in the registry. Furthermore, standardized follow-up visits with wound monitoring, toe pressure measurement and DUS will be scheduled at 6–8 weeks and 12 months after the intervention.

Limitations of this study are the absence of an independent core laboratory and QoL assessments. Instead of an independent core laboratory, we try to minimize (inter-)observer bias by having procedural angiographic parameters and endpoints assessed by 1 person. Other limitations, inherent to a registry, are potential treatment-, attrition- and selection bias. Treatment bias might occur because treatments are not randomly assigned, but chosen based on patient characteristics and physician preference. Any selection bias is observed in this study by maintaining a data log of patients who are not included in the registry.

With an inclusion rate of 400–500 interventions per year, THRILLER aims to provide the largest prospective well phenotyped up-to-date dataset on treatment outcomes of popliteal and infrapopliteal endovascular interventions in the world. To our knowledge, previous dedicated below the knee (BTK) registries included a maximum of 450 endovascular interventions after an inclusion period up to six years [8, 9, 21, 22]. The aim of THRILLER is to generate clinically and scientifically relevant and generalizable data to answer multiple underexplored research questions regarding diagnostics, medication, patient selection, treatment strategies and post intervention follow-up of patients with CLTI that undergo popliteal and infrapopliteal endovascular interventions.

## Supporting information

S1 File (PDF)
