# Supplementary material for: The Dutch chronic lower limb-threatening ischemia registry (THRILLER): A study protocol for popliteal and infrapopliteal endovascular interventions
Source: PLoS One. 2023 Jul 20;18(7):e0288912. doi: 10.1371/journal.pone.0288912 (PMC10358906; doi:10.1371/journal.pone.0288912)
Supplement: S1 File — (PDF) [file pone.0288912.s001.pdf]

Dutch Critical Limb Ischemia Registry

Registry ‘’Crural and pedal pathology’’ - version 248.81

Printed on 19-05-2023 11:42:25 by Michael Nugteren

1. Patient characteristics - Demographics

Number	Question	Answers
1.1	Hospital	◯ UMC Utrecht<br>◯ NWZ Alkmaar<br>◯ Maasstad ziekenhuis Rotterdam<br>◯ Isala Zwolle<br>◯ Sint Antonius Nieuwegein<br>◯ JBZ 's-Hertogenbosch<br>◯ ETZ Tilburg
1.2	Age at date of intervention	[ ] years
1.3	Gender	◯ Female<br>◯ Male
1.4	Height<br>*Notice shown if field's value is smaller than 10: 'Are you sure the length is in centimers?'*	[ ] cm
1.5	Weight	[ ] kg
1.6	BMI	
1.7	Which limb is being treated?	◯ Left<br>◯ Right
1.8	Is the patient already included in the registry for an intervention of a different lesion?	◯ Yes<br>◯ No
1.8.1	***If 'Is the patient already included in the registry for an intervention of a different lesion?' is equal to 'Yes' answer this question:***<br>Is the patient included for an intervention in the other limb or the same limb?	◯ Other limb<br>◯ Other lesion same limb<br>◯ Both
1.8.2	***If 'Is the patient already included in the registry for an intervention of a different lesion?' is equal to 'Yes' answer this question:***<br>How many times was the patient already included in this registry before this inclusion?	◯ 1 time<br>◯ 2 times<br>◯ 3 times<br>◯ 4 times<br>◯ 5 times

1.9	Informed consent	◯ Yes<br>◯ No
1.9.1	***If 'Informed consent' is equal to 'Yes' answer this question:***<br>Informed consent date	☐ ☐ ☐ *(dd-mm-yyyy)*

2. Patient characteristics - Medical history

Number	Question	Answers
2.1	History of smoking	◯ No<br>◯ Yes, former<br>◯ Yes, current<br>◯ Unknown
2.2	History of alcohol abuse	◯ No<br>◯ Yes<br>◯ Unknown
2.3	History of hypertension	◯ No<br>◯ Yes<br>◯ Unknown
2.3.1	***If 'History of hypertension' is equal to 'Yes' answer this question:***<br>Which antihypertensive agents are used?	☐ None<br>☐ Beta-blockers<br>☐ ACE inhibitors<br>☐ Angiotensin II receptor blockers<br>☐ Diuretics<br>☐ Calcium channel blockers<br>☐ Other antihypertensive medication
2.3.1.1	***If 'Which antihypertensive agents are used?' is equal to 'Other antihypertensive medication' answer this question:***<br>Specification other antihypertensive medication	
2.4	History of hyperlipidemia	◯ No<br>◯ Yes, but no lipid-lowering medication<br>◯ Yes, use of statines<br>◯ Yes, use of other lipid-lowering agents<br>◯ Unknown

2.5	History of diabetes	◯ No<br>◯ Yes<br>◯ Unknown
2.5.1	***If 'History of diabetes' is equal to 'Yes' answer this question:***<br>Diabetic medication	◯ None<br>◯ Oral medication<br>◯ Insulin
2.6	Use of specific drugs	☐ No<br>☐ Prednisone<br>☐ Dexamethasone<br>☐ Methotrexate<br>☐ Hydrea (= hydroxyurea)
2.7	History of gout (= jicht)	◯ No<br>◯ Yes<br>◯ Unknown
2.7.1	***If 'History of gout (= jicht)' is equal to 'Yes' answer this question:***<br>Use of colchicine	◯ No<br>◯ Yes, profylactic dose (0.5-1 mg/dag)<br>◯ Yes, only in gout attacks
2.7.1.1	***If 'Use of colchicine' is not equal to 'No' answer this question:***<br>Specification use of colchicine	
2.8	History of cardiovascular disease	◯ No<br>◯ Yes<br>◯ Unknown
2.8.1	***If 'History of cardiovascular disease' is equal to 'Yes' answer this question:***<br>Specification of cardiovascular history	☐ Angina pectoris<br>☐ Myocardial infarction<br>☐ Heart failure (EF ≤ 40% and/or symptoms)<br>☐ Coronary endovascular revascularization (PCI)<br>☐ Coronary open revascularization (CABG)<br>☐ Atrial fibrillation/flutter<br>☐ Pacemaker<br>☐ Defibrillator (ICD)

2.9	Current ischaemic heart disease	◯ No<br>◯ Yes, asymptomatic (but positive stress test)<br>◯ Yes, angina only during strenous or prolonged physical activity<br>◯ Yes, symptoms with everyday living activities<br>◯ Yes, inability to perform any activity without angina or angina at rest
2.10	Left ventricular ejection fraction	[ ] %
2.11	History of cerebrovascular disease	◯ No<br>◯ Yes, TIA (including minor stroke)<br>◯ Yes, CVA<br>◯ Unknown
2.12	History of VTE	◯ No<br>◯ DVT<br>◯ PE (Pulmonary embolism)<br>◯ Both DVT and PE
2.13	History of COPD	◯ No<br>◯ Yes, GOLD 1<br>◯ Yes, GOLD 2<br>◯ Yes, GOLD 3<br>◯ Yes, GOLD 4<br>◯ Yes, unknown GOLD class
2.14	Recent COVID infection	◯ No<br>◯ Yes, for which IC admission<br>◯ Yes, for which hospitalization<br>◯ Yes, but no hospitalization
2.15	History of carotid revascularization	◯ No<br>◯ Yes<br>◯ Unknown
2.16	Antithrombotics baseline	☐ None<br>☐ Acetylsalicylzuur (or carbasalaatcalcium)<br>☐ P2Y12-inhibitor (e.g. clopidogrel, ticagrelor, prasugrel)<br>☐ Dipyridamol<br>☐ Vitamin K antagonist<br>☐ DOAC (e.g. rivaroxaban, dabigatran)<br>☐ Heparins
2.17	If patient uses ascal and DOAC baseline, show DPI	

2.17.1	***If 'If patient uses ascal and DOAC baseline, show DPI' is equal to '1' answer this question:***<br>Is the patient using rivaroxaban 2dd 2.5mg + ascal?	◯ Yes<br>◯ No
2.18	Specification (and indication) antithrombotics baseline	
2.19	***If 'Hospital' is equal to 'UMC Utrecht' answer this question:***<br>Did patient already test on VerifyNow testing	☐ No<br>☐ Yes, the clopidogrel test<br>☐ Yes, the aspirin test
2.19.1	***If 'Did patient already test on VerifyNow testing' is equal to 'Yes, the clopidogrel test' answer this question:***<br>According to VerifyNow clopidogrel response	◯ Good response<br>◯ Bad response
2.19.2	***If 'Did patient already test on VerifyNow testing' is equal to 'Yes, the aspirin test' answer this question:***<br>According to VerifyNow aspirin response	◯ Good response<br>◯ Bad response
2.20	History of bleeding event (read information)	☐ No<br>☐ Transfusion during index hospitalisation<br>☐ Prior diagnosis of coagulopathy<br>☐ Prior primary diagnosis of major bleeding
2.20.1	***If 'History of bleeding event (read information)' is not equal to 'No' answer this question:***<br>Specification history of bleeding	
2.21	Renal insufficiency	☐ No<br>☐ Yes (GFR < 30)<br>☐ Yes, end stage renal disease (GFR < 15)<br>☐ Yes, dialysis dependent<br>☐ Unknown<br>☐ Status after kidney transplant
2.22	History of malignancy	◯ Yes<br>◯ No

2.22.1	***If 'History of malignancy' is equal to 'Yes' answer this question:***<br>What type(s) of malignancy?	☐ Lung malignancy<br>☐ Breast malignancy<br>☐ Prostate malignancy<br>☐ Colorectal malignancy<br>☐ Bladder malignancy<br>☐ Renal malignancy<br>☐ Pancreatic malignancy<br>☐ Hematologic malignancy<br>☐ Skin malignancy<br>☐ Gynecologic malignancy<br>☐ Other
2.23.1	***If 'Current treatment for malignancy' is equal to 'Yes' answer this question:***<br>Specification type of malignancy	
2.22.2	***If 'History of malignancy' is equal to 'Yes' answer this question:***<br>Current treatment for malignancy	○ No<br>○ Yes<br>○ Unknown
2.22.2.2	***If 'Current treatment for malignancy' is equal to 'Yes' answer this question:***<br>What type of current malignancy?	○ Lung malignancy<br>○ Breast malignancy<br>○ Prostate malignancy<br>○ Colorectal malignancy<br>○ Bladder malignancy<br>○ Renal malignancy<br>○ Pancreatic malignancy<br>○ Hematologic malignancy<br>○ Skin malignancy<br>○ Gynecologic malignancy<br>○ Other
2.22.2.3	***If 'Current treatment for malignancy' is equal to 'Yes' answer this question:***<br>Specification type of current malignancy	
2.22.2.4	***If 'Current treatment for malignancy' is equal to 'Yes' answer this question:***<br>What current treatment for malignancy	☐ None<br>☐ Chemotherapy<br>☐ Radiation therapy<br>☐ Immunotherapy<br>☐ Surgery

2.23	History of dementia	◯ No<br>◯ Yes<br>◯ Unknown
2.23.1	***If 'History of dementia' is equal to 'Yes' answer this question:***<br>Vascular dementia?	◯ Yes<br>◯ No
2.23.2	***If 'History of dementia' is equal to 'Yes' answer this question:***<br>Grade of dementia	◯ CDR 1<br>◯ CDR 2<br>◯ CDR 3<br>◯ Unknown
2.24	ASA grade	◯ ASA 1: normal healthy patient<br>◯ ASA 2: mild systemic disease<br>◯ ASA 3: severe systematic disease<br>◯ ASA 4: severe systematic disease that is a constant threat to life<br>◯ ASA 5: moribund patient who is not expected to survive without the operation
2.25	Living status	◯ Lives independently<br>◯ Lives home with home care (at least once a day)<br>◯ Lives in a nursing home<br>◯ Unknown
2.26	Functional status	◯ Moves outdoors independently<br>◯ Moves outdoors with walking aids<br>◯ Moves indoors with or without walking aids<br>◯ Minimal ambulant/wheelchair: uses legs for small transfers<br>◯ Bed-bound<br>◯ Uknown

3. Patient characteristics - PAD history

Number	Question	Answers
3.1	Previous hospital stay for PAD	◯ Yes<br>◯ No

3.2	Previous PAD revascularizations	◯ No<br>◯ Yes<br>◯ Unknown
3.2.1	***If 'Previous PAD revascularizations' is equal to 'Yes' answer this question:***<br>Which limb	◯ Ipsilateral limb<br>◯ Contralateral limb<br>◯ Both
3.2.1.1	***If 'Which limb' is not equal to 'Contralateral limb' answer this question:***<br>Type of intervention same limb	☐ Endovascular<br>☐ Femoral endarterectomy<br>☐ Bypass<br>☐ Limflow procedure<br>☐ None
3.2.1.2	***If 'Which limb' is not equal to 'Contralateral limb' answer this question:***<br>Number of previous revascularizations same limb	◯ 0<br>◯ 1<br>◯ 2<br>◯ 3<br>◯ 4<br>◯ 5
3.2.1.3	***If 'Which limb' is not equal to 'Contralateral limb' answer this question:***<br>Which arteries are revascularized in previous revascularization(s) same limb?	☐ Aorta<br>☐ AIC<br>☐ AIE<br>☐ AFC<br>☐ AFS<br>☐ A. poplitea (P1)<br>☐ A. poplitea (P2-P3)<br>☐ ATA<br>☐ Truncus tibioperonealis<br>☐ A. peroneale<br>☐ ATP<br>☐ Pedal arteries
3.2.1.4	***If 'Which limb' is not equal to 'Ipsilateral limb' answer this question:***<br>Type of intervention other limb	☐ Endovascular<br>☐ Femoral endarterectomy<br>☐ Bypass<br>☐ Limflow procedure<br>☐ None

3.2.1.5	***If 'Which limb' is not equal to 'Ipsilateral limb' answer this question:*** Number of previous revascularizations other limb	◯ 0<br>◯ 1<br>◯ 2<br>◯ 3<br>◯ 4<br>◯ 5
3.2.1.6	***If 'Which limb' is not equal to 'Ipsilateral limb' answer this question:*** Which arteries are revascularized in previous revascularization(s) other limb?	☐ Aorta<br>☐ AIC<br>☐ AIE<br>☐ AFC<br>☐ AFS<br>☐ A. poplitea (P1)<br>☐ A. poplitea (P2-P3)<br>☐ ATA<br>☐ Truncus tibioperonealis<br>☐ A. peroneale<br>☐ ATP<br>☐ Pedal arteries
3.2.1.7	***If 'Which limb' is not equal to 'Contralateral limb' answer this question:*** Similar lesion/location as current intervention	☐ No<br>☐ Yes<br>☐ Unknown
3.2.1.7.1	***If 'Similar lesion/location as current intervention' is equal to 'Yes' answer this question:*** Date of previous revascularization same lesion (1)	[ ] [ ] [ ] *(dd-mm-yyyy)*
3.2.1.7.2	***If 'Similar lesion/location as current intervention' is equal to 'Yes' answer this question:*** Type of intervention same lesion (1)	☐ Endovascular<br>☐ Femoral endarterectomy<br>☐ Bypass<br>☐ Limflow procedure<br>☐ None
3.2.1.7.2.1	***If 'Type of intervention same lesion (1)' is equal to 'Endovascular' answer this question:*** Type of endovascular intervention (1)	☐ PTA<br>☐ BMS<br>☐ DCB<br>☐ DES<br>☐ Atherectomy<br>☐ Other<br>☐ Unknown

3.2.1.7.3	***If 'Similar lesion/location as current intervention' is equal to 'Yes' answer this question:***<br>Which arteries are involved in revascularization same lesion (1)	☐ A. poplitea<br>☐ A. tibialis anterior (ATA)<br>☐ Truncus tibioperonealis<br>☐ A. peronea<br>☐ A. tibialis posterior (ATP)<br>☐ Pedal arteries
3.2.1.7.4	***If 'Similar lesion/location as current intervention' is equal to 'Yes' answer this question:***<br>More previous interventions same lesion?	◯ No<br>◯ Yes, 1 more<br>◯ Yes, 2 more
3.2.1.7.4.1	***If 'More previous interventions same lesion?' is not equal to 'No' answer this question:***<br>Date of previous revascularization same lesion (2)	☐ ☐ ☐ *(dd-mm-yyyy)*
3.2.1.7.4.2	***If 'More previous interventions same lesion?' is not equal to 'No' answer this question:***<br>Type of intervention same lesion (2)	☐ Endovascular<br>☐ Femoral endarterectomy<br>☐ Bypass<br>☐ Limflow procedure<br>☐ None
3.2.1.7.4.2.1	***If 'Type of intervention same lesion (2)' is equal to 'Endovascular' answer this question:***<br>Type of endovascular intervention (2)	☐ PTA<br>☐ BMS<br>☐ DCB<br>☐ DES<br>☐ Atherectomy<br>☐ Other<br>☐ Unknown
3.2.1.7.4.3	***If 'More previous interventions same lesion?' is not equal to 'No' answer this question:***<br>Which arteries are involved in revascularization same lesion (2)	☐ A. poplitea<br>☐ A. tibialis anterior (ATA)<br>☐ Truncus tibioperonealis<br>☐ A. peronea<br>☐ A. tibialis posterior (ATP)<br>☐ Pedal arteries
3.2.1.7.4.4	***If 'More previous interventions same lesion?' is equal to 'Yes, 2 more' answer this question:***<br>Date of previous revascularization same lesion (3)	☐ ☐ ☐ *(dd-mm-yyyy)*
3.2.1.7.4.5	***If 'More previous interventions same lesion?' is equal to 'Yes, 2 more' answer this question:***<br>Type of intervention same lesion (2)	☐ Endovascular<br>☐ Femoral endarterectomy<br>☐ Bypass<br>☐ Limflow procedure<br>☐ None

3.2.1.7.4.5.1	***If 'Type of intervention same lesion (2)' is equal to 'Endovascular' answer this question:***<br>Type of endovascular intervention (3)	☐ PTA<br>☐ BMS<br>☐ DCB<br>☐ DES<br>☐ Atherectomy<br>☐ Other<br>☐ Unknown
3.2.1.7.4.6	***If 'More previous interventions same lesion?' is equal to 'Yes, 2 more' answer this question:***<br>Which arteries are involved in revascularization same lesion (3)	☐ A. poplitea<br>☐ A. tibialis anterior (ATA)<br>☐ Truncus tibioperonealis<br>☐ A. peronea<br>☐ A. tibialis posterior (ATP)<br>☐ Pedal arteries
3.2.2	***If 'Previous PAD revascularizations' is equal to 'Yes' answer this question:***<br>Comments on previous revascularizations	
3.3	Previous amputations	○ No<br>○ Yes<br>○ Unknown
3.3.1	***If 'Previous amputations' is equal to 'Yes' answer this question:***<br>Amputations in which limb?	○ Ipsilateral limb<br>○ Contralateral limb<br>○ Both
3.3.1.1	***If 'Amputations in which limb?' is not equal to 'Contralateral limb' answer this question:***<br>What amputation(s) on ipsilateral limb	☐ Dig 1<br>☐ Dig 2<br>☐ Dig 3<br>☐ Dig 4<br>☐ Dig 5<br>☐ Forefoot<br>☐ Ankle
3.3.1.2	***If 'Amputations in which limb?' is not equal to 'Contralateral limb' answer this question:***<br>When was the most recent amputation on the ipsilateral limb?	*(dd-mm-yyyy)*

3.3.1.3	***If 'Amputations in which limb?' is not equal to 'Ipsilateral limb' answer this question:***<br>What amputation(s) on contralateral limb	☐ Dig 1<br>☐ Dig 2<br>☐ Dig 3<br>☐ Dig 4<br>☐ Dig 5<br>☐ Forefoot<br>☐ Ankle<br>☐ Below the knee<br>☐ Through the knee<br>☐ Above the knee
3.3.1.4	***If 'Amputations in which limb?' is not equal to 'Ipsilateral limb' answer this question:***<br>When was the most recent amputation on the contralateral limb?	*(dd-mm-yyyy)*
3.3.2	***If 'Previous amputations' is equal to 'Yes' answer this question:***<br>Comments on previous amputations	

4. Baseline - Ankle-brachial index

Number	Question	Answers
4.1	Date most recent ABI	*(dd-mm-yyyy)*
4.2	Blood pressure arm	
4.2.1	***If 'Blood pressure arm' is smaller than '300' answer this question:***<br>Arteries ankle in which ankle pressure is measured	☐ ATP<br>☐ ADP<br>☐ Unknown
4.2.1.1	***If 'Arteries ankle in which ankle pressure is measured' is equal to 'ATP' answer this question:***<br>Blood pressure ATP	
4.2.1.1.1	***If 'Blood pressure ATP' is greater or equal than '1' answer this question:***<br>ABI based on ATP baseline	
4.2.1.2	***If 'Arteries ankle in which ankle pressure is measured' is equal to 'ADP' answer this question:***<br>Blood pressure ADP	
4.2.1.2.1	***If 'Blood pressure ADP' is greater or equal than '1' answer this question:***<br>ABI based on ADP baseline	

4.3	Highest blood pressure in ankle	
4.3.1	***If 'Highest blood pressure in ankle' is greater or equal than '1' answer this question:***<br>Highest ABI in ankle	
4.3.2	***If 'Highest blood pressure in ankle' is smaller than '5' answer this question:***<br>Highest ABI fill in yourself	
4.3.3	***If 'Highest blood pressure in ankle' is smaller than '5' answer this question:***<br>Reason no ankle pressure baseline	◯ Not compressible<br>◯ Not measured<br>◯ Other
4.3.3.1	***If 'Reason no ankle pressure baseline' is equal to 'Other' answer this question:***<br>Specification reason absence ankle pressure	
4.4	Toe pressure	
4.4.1	***If 'Toe pressure' is greater or equal than '1' answer this question:***<br>TBI baseline	
4.4.2	***If 'Toe pressure' is greater than '-1' answer this question:***<br>Toe pressure curve	
4.4.3	***If 'Toe pressure' is greater than '-1' answer this question:***<br>Toe pressure acceleration time	
4.4.4	***If 'Toe pressure' is greater than '-1' answer this question:***<br>Ankle pressure curve (optional)	
4.4.5	***If 'Toe pressure' is greater than '-1' answer this question:***<br>Ankle (ATP) pressure acceleration time	
4.4.6	***If 'Toe pressure' is greater than '-1' answer this question:***<br>Ankle (ADP) pressure acceleration time	

5. Baseline - Radiological characteristics

Number	Question	Answers
5.1	Imaging modalities available	☐ None<br>☐ Duplex ultrasound<br>☐ MRA<br>☐ CTA<br>☐ Angiography

5.2	Was an X-ray of the foot taken pre-operatively?	◯ No<br>◯ Yes<br>◯ Other
5.2.1	***If 'Was an X-ray of the foot taken pre-operatively?' is equal to 'Yes' answer this question:***<br>X-ray foot useful for calcium scoring?	◯ Yes<br>◯ No
5.2.2	***If 'Was an X-ray of the foot taken pre-operatively?' is equal to 'Other' answer this question:***<br>Text box X-ray foot pre-operatively	
5.1.1	***If 'Imaging modalities available' is equal to 'Duplex ultrasound' answer this question:***<br>Date duplex baseline	*(dd-mm-yyyy)*
5.1.2	***If 'Imaging modalities available' is equal to 'MRA' answer this question:***<br>Date MRA baseline	*(dd-mm-yyyy)*
5.1.3	***If 'Imaging modalities available' is equal to 'CTA' answer this question:***<br>Date CTA baseline	*(dd-mm-yyyy)*
5.1.4	***If 'Imaging modalities available' is equal to 'Angiography' answer this question:***<br>Date angiography baseline	*(dd-mm-yyyy)*
5.3	In which segments is a significant stenosis/occlusion present?	☐ A. poplitea<br>☐ A. tibialis anterior (ATA)<br>☐ Truncus tibioperonealis<br>☐ A. peronea<br>☐ A. tibialis posterior (ATP)<br>☐ Pedal arteries
5.3.1	***If 'In which segments is a significant stenosis/occlusion present?' is equal to 'A. poplitea' answer this question:***<br>Significant stenosis/occlusions poplitea	☐ P1 (above the upper border of patella)<br>☐ P2 (above joint line of knee)<br>☐ P3 (under joint line of knee)
5.3.2	***If 'In which segments is a significant stenosis/occlusion present?' is equal to 'A. tibialis anterior (ATA)' answer this question:***<br>Significant stenosis/occlusions ATA	☐ Proximal ATA<br>☐ Mid ATA<br>☐ Distal ATA
5.3.3	***If 'In which segments is a significant stenosis/occlusion present?' is equal to 'A. peronea' answer this question:***<br>Significant stenosis/occlusions peroneal artery	☐ Proximal peronea<br>☐ Mid peronea<br>☐ Distal peronea

5.3.4	***If 'In which segments is a significant stenosis/occlusion present?' is equal to 'A. tibialis posterior (ATP)' answer this question:***<br>Significant stenosis/occlusions ATP	☐ Proximal ATP<br>☐ Mid ATP<br>☐ Distal ATP
5.3.5	***If 'In which segments is a significant stenosis/occlusion present?' is equal to 'Pedal arteries' answer this question:***<br>Significant stenosis/occlusions pedal arteries	☐ Arteria dorsalis pedis<br>☐ Arteria plantaris lateralis<br>☐ Arteria plantaris medialis<br>☐ Plantar arterial arch
5.3.1.1	***If 'Significant stenosis/occlusions poplitea' is equal to 'P1 (above the upper border of patella)' answer this question:***<br>P1 lesion degree of stenosis based on radiologic imaging/angiography	○ Stenosis non-significant (<50%)<br>○ Stenosis significant (≥50%)<br>○ Total occlusion
5.3.1.2	***If 'Significant stenosis/occlusions poplitea' is equal to 'P2 (above joint line of knee)' answer this question:***<br>P2 lesion degree of stenosis based on radiologic imaging/angiography	○ Stenosis non-significant (<50%)<br>○ Stenosis significant (≥50%)<br>○ Total occlusion
5.3.1.3	***If 'Significant stenosis/occlusions poplitea' is equal to 'P3 (under joint line of knee)' answer this question:***<br>P3 lesion degree of stenosis based on radiologic imaging/angiography	○ Stenosis non-significant (<50%)<br>○ Stenosis significant (≥50%)<br>○ Total occlusion
5.3.2.1	***If 'Significant stenosis/occlusions ATA' is equal to 'Proximal ATA' answer this question:***<br>Proximal ATA lesion degree of stenosis based on radiologic imaging/angiography	○ Stenosis non-significant (<50%)<br>○ Stenosis significant (≥50%)<br>○ Total occlusion
5.3.2.2	***If 'Significant stenosis/occlusions ATA' is equal to 'Mid ATA' answer this question:***<br>Mid ATA lesion degree of stenosis based on radiologic imaging/angiography	○ Stenosis non-significant (<50%)<br>○ Stenosis significant (≥50%)<br>○ Total occlusion
5.3.2.3	***If 'Significant stenosis/occlusions ATA' is equal to 'Distal ATA' answer this question:***<br>Distal ATA lesion degree of stenosis based on radiologic imaging/angiography	○ Stenosis non-significant (<50%)<br>○ Stenosis significant (≥50%)<br>○ Total occlusion
5.3.6	***If 'In which segments is a significant stenosis/occlusion present?' is equal to 'Truncus tibioperonealis' answer this question:***<br>TTP lesion degree of stenosis based on radiologic imaging/angiography	○ Stenosis non-significant (<50%)<br>○ Stenosis significant (≥50%)<br>○ Total occlusion
5.3.3.1	***If 'Significant stenosis/occlusions peroneal artery' is equal to 'Proximal peronea' answer this question:***<br>Proximal peronea lesion degree of stenosis based on radiologic imaging/angiography	○ Stenosis non-significant (<50%)<br>○ Stenosis significant (≥50%)<br>○ Total occlusion

5.3.3.2	***If 'Significant stenosis/occlusions peroneal artery' is equal to 'Mid peronea' answer this question:***<br>Mid peronea lesion degree of stenosis based on radiologic imaging/angiography	◯ Stenosis non-significant (<50%)<br>◯ Stenosis significant (≥50%)<br>◯ Total occlusion
5.3.3.3	***If 'Significant stenosis/occlusions peroneal artery' is equal to 'Distal peronea' answer this question:***<br>Distal peronea lesion degree of stenosis based on radiologic imaging/angiography	◯ Stenosis non-significant (<50%)<br>◯ Stenosis significant (≥50%)<br>◯ Total occlusion
5.3.4.1	***If 'Significant stenosis/occlusions ATP' is equal to 'Proximal ATP' answer this question:***<br>Proximal ATP lesion degree of stenosis based on radiologic imaging/angiography	◯ Stenosis non-significant (<50%)<br>◯ Stenosis significant (≥50%)<br>◯ Total occlusion
5.3.4.2	***If 'Significant stenosis/occlusions ATP' is equal to 'Mid ATP' answer this question:***<br>Mid ATP lesion degree of stenosis based on radiologic imaging/angiography	◯ Stenosis non-significant (<50%)<br>◯ Stenosis significant (≥50%)<br>◯ Total occlusion
5.3.4.3	***If 'Significant stenosis/occlusions ATP' is equal to 'Distal ATP' answer this question:***<br>Distal ATP lesion degree of stenosis based on radiologic imaging/angiography	◯ Stenosis non-significant (<50%)<br>◯ Stenosis significant (≥50%)<br>◯ Total occlusion
5.3.5.1	***If 'Significant stenosis/occlusions pedal arteries' is equal to 'Arteria dorsalis pedis' answer this question:***<br>ADP lesion degree of stenosis based on radiologic imaging/angiography	◯ Stenosis non-significant (<50%)<br>◯ Stenosis significant (≥50%)<br>◯ Total occlusion
5.3.5.2	***If 'Significant stenosis/occlusions pedal arteries' is equal to 'Arteria plantaris lateralis' answer this question:***<br>APL lesion degree of stenosis based on radiologic imaging/angiography	◯ Stenosis non-significant (<50%)<br>◯ Stenosis significant (≥50%)<br>◯ Total occlusion
5.3.5.3	***If 'Significant stenosis/occlusions pedal arteries' is equal to 'Arteria plantaris medialis' answer this question:***<br>APM lesion degree of stenosis based on radiologic imaging/angiography	◯ Stenosis non-significant (<50%)<br>◯ Stenosis significant (≥50%)<br>◯ Total occlusion
5.4	Number of target lesions	◯ 1<br>◯ 2<br>◯ 3<br>◯ 4

5.5	Target lesion location (1)	☐ A. poplitea (P1)<br>☐ A. poplitea (P2)<br>☐ A. poplitea (P3)<br>☐ Truncus tibioperonealis<br>☐ ATA proximal<br>☐ ATA mid<br>☐ ATA distal<br>☐ A. peronea proximal<br>☐ A. peronea mid<br>☐ A. peronea distal<br>☐ ATP proximal<br>☐ ATP mid<br>☐ ATP distal<br>☐ A. dorsalis pedis<br>☐ A. plantaris lateralis<br>☐ A. plantaris medialis<br>☐ Plantar arterial arch
5.6	Target lesion degree of stenosis	○ Stenosis non-significant (<50%)<br>○ Stenosis significant (≥50%)<br>○ Total occlusion
5.7	Target lesion de novo, instent or in bypass? (1)	○ De novo lesion<br>○ Restenosis/-occlusion<br>○ Instent restenosis/-occlusion<br>○ In bypass restenosis/-occlusion<br>○ Instent in bypass restenosis/-occlusion
5.4.1	***If 'Number of target lesions' is not equal to '1' answer this question:***<br>Target lesion location (2)	☐ A. poplitea (P1)<br>☐ A. poplitea (P2)<br>☐ A. poplitea (P3)<br>☐ Truncus tibioperonealis<br>☐ ATA proximal<br>☐ ATA mid<br>☐ ATA distal<br>☐ A. peronea proximal<br>☐ A. peronea mid<br>☐ A. peronea distal<br>☐ ATP proximal<br>☐ ATP mid<br>☐ ATP distal<br>☐ A. dorsalis pedis<br>☐ A. plantaris lateralis<br>☐ A. plantaris medialis<br>☐ Plantar arterial arch

5.4.2	***If 'Number of target lesions' is not equal to '1' answer this question:***<br>Target lesion degree of stenosis based on radiologic imaging (2)	◯ Stenosis non-significant (<50%)<br>◯ Stenosis significant (≥50%)<br>◯ Total occlusion
5.4.3	***If 'Number of target lesions' is not equal to '1' answer this question:***<br>Target lesion de novo, instent or in bypass? (2)	◯ De novo lesion<br>◯ Restenosis/-occlusion<br>◯ Instent restenosis/-occlusion<br>◯ In bypass restenosis/-occlusion<br>◯ Instent in bypass restenosis/-occlusion
5.8	If 3-4 TL, show next questions	
5.8.1	***If 'If 3-4 TL, show next questions' is equal to '1' answer this question:***<br>Target lesion location (3)	☐ A. poplitea (P1)<br>☐ A. poplitea (P2)<br>☐ A. poplitea (P3)<br>☐ Truncus tibioperonealis<br>☐ ATA proximal<br>☐ ATA mid<br>☐ ATA distal<br>☐ A. peronea proximal<br>☐ A. peronea mid<br>☐ A. peronea distal<br>☐ ATP proximal<br>☐ ATP mid<br>☐ ATP distal<br>☐ A. dorsalis pedis<br>☐ A. plantaris lateralis<br>☐ A. plantaris medialis<br>☐ Plantar arterial arch
5.8.2	***If 'If 3-4 TL, show next questions' is equal to '1' answer this question:***<br>Target lesion degree of stenosis based on radiologic imaging (3)	◯ Stenosis non-significant (<50%)<br>◯ Stenosis significant (≥50%)<br>◯ Total occlusion
5.8.3	***If 'If 3-4 TL, show next questions' is equal to '1' answer this question:***<br>Target lesion de novo, instent or in bypass? (3)	◯ De novo lesion<br>◯ Restenosis/-occlusion<br>◯ Instent restenosis/-occlusion<br>◯ In bypass restenosis/-occlusion<br>◯ Instent in bypass restenosis/-occlusion

5.4.4	***If 'Number of target lesions' is equal to '4' answer this question:***<br>Target lesion location (4)	☐ A. poplitea (P1)<br>☐ A. poplitea (P2)<br>☐ A. poplitea (P3)<br>☐ Truncus tibioperonealis<br>☐ ATA proximal<br>☐ ATA mid<br>☐ ATA distal<br>☐ A. peronea proximal<br>☐ A. peronea mid<br>☐ A. peronea distal<br>☐ ATP proximal<br>☐ ATP mid<br>☐ ATP distal<br>☐ A. dorsalis pedis<br>☐ A. plantaris lateralis<br>☐ A. plantaris medialis<br>☐ Plantar arterial arch
5.4.5	***If 'Number of target lesions' is equal to '4' answer this question:***<br>Target lesion degree of stenosis based on radiologic imaging (4)	○ Stenosis non-significant (<50%)<br>○ Stenosis significant (≥50%)<br>○ Total occlusion
5.4.6	***If 'Number of target lesions' is equal to '4' answer this question:***<br>Target lesion de novo, instent or in bypass? (4)	○ De novo lesion<br>○ Restenosis/-occlusion<br>○ Instent restenosis/-occlusion<br>○ In bypass restenosis/-occlusion<br>○ Instent in bypass restenosis/-occlusion

6. Baseline - Limb staging (including WIfI)

Number	Question	Answers
6.1	Rest pain baseline	○ Yes<br>○ No
6.2	Number of ulcers baseline	○ 0<br>○ 1<br>○ 2<br>○ 3<br>○ 4

6.2.1	***If 'Number of ulcers baseline' is not equal to '0' answer this question:***<br>Location ulcers baseline	☐ Calf<br>☐ Pretibial<br>☐ Ankle<br>☐ Foot<br>☐ Toe(s)<br>☐ Heel<br>☐ Unknown
6.2.2	***If 'Number of ulcers baseline' is not equal to '0' answer this question:***<br>Pathofysiology of ulcers	☐ None<br>☐ Peripheral polyneuropathy<br>☐ Chronic venous insufficiency<br>☐ Traumatic<br>☐ Iatrogenic (including podiatric treatment)<br>☐ Gout (= jicht)<br>☐ Other arthritis<br>☐ Martorell ulcer<br>☐ Drug-induced (e.g. hydrea, prednisone or methotrexate)<br>☐ Other (please explain)
6.2.3	***If 'Number of ulcers baseline' is not equal to '0' answer this question:***<br>Pathofysiology text box	
6.3	Fontaine classification	○ Fontaine 1 (asymptomatic)<br>○ Fontaine 2a (pain free walking distance > 200m)<br>○ Fontaine 2b (pain free walking distance < 200m)<br>○ Fontaine 3 (rest pain)<br>○ Fontaine 4 (ulceration or gangrene)
6.4	Rutherford classification	○ 0 (asymptomatic)<br>○ 1 (mild claudation)<br>○ 2 (moderate claudication)<br>○ 3 (severe claudication)<br>○ 4 (rest pain)<br>○ 5 (minor tissue loss)<br>○ 6 (major tissue loss)
6.3.1	***If 'Fontaine classification' is equal to 'Fontaine 4 (ulceration or gangrene)' answer this question:***<br>Gangrene baseline	○ Yes<br>○ No
6.5	If CLTI, than...	

6.5.1	***If 'If CLTI, than...' is equal to '1' answer this question:***<br>Wound (WIfI) baseline	◯ 0: No ulcer and no gangrene<br>◯ 1: Small ulcer and no gangrene<br>◯ 2: Deep ulcer or gangrene limited to toes<br>◯ 3: Extensive ulcer or extensive gangrene
6.5.2	***If 'If CLTI, than...' is equal to '1' answer this question:***<br>Ischemia (WIfI) baseline	◯ 0: > 60 mmHg<br>◯ 1: 40 - 59 mmHg<br>◯ 2: 30 - 39 mmHg<br>◯ 3: < 30 mmHg
6.5.3	***If 'If CLTI, than...' is equal to '1' answer this question:***<br>Ischemia (WIfI) based on..	◯ Toe pressure<br>◯ ABI (or ankle pressure)<br>◯ TcPO2
6.5.4	***If 'If CLTI, than...' is equal to '1' answer this question:***<br>Foot Infection (WIfI) baseline	◯ 0: Noninfected<br>◯ 1: Mild (< 2 cm cellulitis)<br>◯ 2: Moderate (> 2 cm cellulitis / purulence)<br>◯ 3: Severe (systemic response / sepsis)
6.5.5	***If 'If CLTI, than...' is equal to '1' answer this question:***<br>WIfI assignment amputation image	
6.5.6	***If 'If CLTI, than...' is equal to '1' answer this question:***<br>WIfI assignment amputation risk baseline	◯ 1: Very low<br>◯ 2: Low<br>◯ 3: Moderate<br>◯ 4: High
6.5.7	***If 'If CLTI, than...' is equal to '1' answer this question:***<br>WIfI assignment benefit revascularization image	
6.5.8	***If 'If CLTI, than...' is equal to '1' answer this question:***<br>WIfI assignment benefit revascularization baseline	◯ 1: Very low<br>◯ 2: Low<br>◯ 3: Moderate<br>◯ 4: High
6.2.4	***If 'Number of ulcers baseline' is not equal to '0' answer this question:***<br>Explanation ulcers baseline	

7. Baseline - Lab values

Number	Question	Answers

7.1	Hemoglobin level baseline	mmol/L
7.2	Hematocrit level baseline	%
7.3	Leucocytes level baseline	x 10^9/L
7.3.1	***If 'Leucocytes level baseline' is greater than '0' answer this question:*** Lymphocytes level baseline	
7.3.2	***If 'Leucocytes level baseline' is greater than '0' answer this question:*** Neutrophils level baseline	
7.4	CRP level baseline	mg/L
7.5	Creatinine level baseline	umol/L
7.6	MDRD GFR baseline	
7.7	Total cholesterol level baseline	mmol/L
7.8	LDL value baseline	mmol/L
7.9	HDL value baseline	mmol/L
7.10	Triglycerides level	mmol/L
7.11	HbA1c level	mmol/mol
7.12	Fluid electrolyte and acid-base balance disorders (with clinical importance)	◯ Yes<br>◯ No

8. Intervention - Intervention general and device characteristics

Number	Question	Answers
8.1	Date of intervention	*(dd-mm-yyyy)*
8.2	Is the revascularization performed after an urgent admission to the hospital (outpatient clinic or ER)?	◯ No, elective surgery.<br>◯ Yes, the admission was via the ER or outpatient clinic.<br>◯ Unknown
8.2.1	***If 'Is the revascularization performed after an urgent admission to the hospital (outpatient clinic or ER)?' is not equal to 'No, elective surgery.' answer this question:*** Time of intervention after admission	hours

8.3	Type of anesthesia	◯ General anesthesia<br>◯ Spinal anesthesia<br>◯ Local anesthesia
8.4	Type of angiologist	☐ Vascular surgeon<br>☐ Interventional radiologist<br>☐ Other
8.4.1	***If 'Type of angiologist' is equal to 'Other' answer this question:***<br>Specification type of angiologist	
8.5	Access site	☐ No possible access<br>☐ Femoral retrograde<br>☐ Femoral anterograde<br>☐ Popliteal<br>☐ Dorsal pedal<br>☐ Posterior tibial<br>☐ Arm
8.6	Largest sheath size	Fr
8.7	Heparin amount administered at start of procedure<br>*Notice shown if field's value is smaller than 100: 'Are you sure you filled in the correct amount?'*	IU
8.8	Extra administrations of heparin after initial dose	◯ 0<br>◯ 1<br>◯ 2<br>◯ 3<br>◯ 4
8.8.1	***If 'Extra administrations of heparin after initial dose' is not equal to '0' answer this question:***<br>Heparin amount administered at second administration<br>*Notice shown if field's value is smaller than 100: 'Are you sure you filled in the correct amount?'*	IU
8.9	If more than 2 heparin administrations, show adm 3	
8.9.1	***If 'If more than 2 heparin administrations, show adm 3' is equal to '1' answer this question:***<br>Heparin amount administered at third administration<br>*Notice shown if field's value is smaller than 100: 'Are you sure you filled in the correct amount?'*	IU
8.10	If more than 3 heparin administrations, show adm 4	

8.10.1	***If 'If more than 3 heparin administrations, show adm 4' is equal to '1' answer this question:***<br>Heparin amount administered at fourth administration<br>*Notice shown if field's value is smaller than 100: 'Are you sure you filled in the correct amount?'*	[ ] IU
8.8.2	***If 'Extra administrations of heparin after initial dose' is equal to '4' answer this question:***<br>Heparin amount administered at fifth administration<br>*Notice shown if field's value is smaller than 100: 'Are you sure you filled in the correct amount?'*	[ ] IU
8.11	Number of BTK lesions treated	○ 1<br>○ 2<br>○ 3<br>○ 4
8.12	Location lesion 1	☐ A. poplitea (P1)<br>☐ A. poplitea (P2)<br>☐ A. poplitea (P3)<br>☐ Truncus tibioperonealis<br>☐ ATA proximal<br>☐ ATA mid<br>☐ ATA distal<br>☐ A. peronea proximal<br>☐ A. peronea mid<br>☐ A. peronea distal<br>☐ ATP proximal<br>☐ ATP mid<br>☐ ATP distal<br>☐ A. dorsalis pedis<br>☐ A. plantaris lateralis<br>☐ A. plantaris medialis<br>☐ Plantar arterial arch
8.12.1	***If 'Location lesion 1' is equal to 'A. poplitea (P1)' answer this question:***<br>Continuous AFS-POP lesion 1?	○ Yes<br>○ No
8.13	Is lesion 1 continuous POP-crural or crural-pedal?	☐ None<br>☐ POP-crural<br>☐ Crural-pedal
8.14	Bifurcation or trifurcation lesion 1?	○ No<br>○ Bifurcation lesion<br>○ Trifurcation lesion

8.15	Does lesion 1 consist of multiple stenoses/occlusions?	◯ Yes<br>◯ No
8.16	Degree of stenosis based on angiography lesion 1	◯ Stenosis non-significant (<50%)<br>◯ Stenosis significant (≥50%)<br>◯ Total occlusion
8.17	Lesion length based on angiography lesion 1	______ mm
8.12.1.1	***If 'Continuous AFS-POP lesion 1?' is equal to 'Yes' answer this question:***<br>Total lesion length lesion 1 (including femoral artery)	______
8.13.1	***If 'Is lesion 1 continuous POP-crural or crural-pedal?' is equal to 'POP-crural' answer this question:***<br>Popliteal lesion length lesion 1	______ mm
8.13.2	***If 'Is lesion 1 continuous POP-crural or crural-pedal?' is equal to 'POP-crural' answer this question:***<br>Popliteal lesion degree of stenosis lesion 1	◯ Stenosis non-significant (<50%)<br>◯ Stenosis significant (≥50%)<br>◯ Total occlusion
8.13.3	***If 'Is lesion 1 continuous POP-crural or crural-pedal?' is not equal to 'None' answer this question:***<br>Crural lesion length lesion 1	______ mm
8.13.4	***If 'Is lesion 1 continuous POP-crural or crural-pedal?' is not equal to 'None' answer this question:***<br>Crural lesion degree of stenosis lesion 1	◯ Stenosis non-significant (<50%)<br>◯ Stenosis significant (≥50%)<br>◯ Total occlusion
8.13.5	***If 'Is lesion 1 continuous POP-crural or crural-pedal?' is equal to 'Crural-pedal' answer this question:***<br>Pedal lesion length lesion 1	______ mm
8.13.6	***If 'Is lesion 1 continuous POP-crural or crural-pedal?' is equal to 'Crural-pedal' answer this question:***<br>Pedal lesion degree of stenosis lesion 1	◯ Stenosis non-significant (<50%)<br>◯ Stenosis significant (≥50%)<br>◯ Total occlusion
8.18	Endovascular techniques lesion 1	☐ PTA<br>☐ PTA/stent<br>☐ DCB<br>☐ DES<br>☐ Shockwave balloon (intravascular lithotripsy)<br>☐ Atherectomy<br>☐ Local thrombolysis<br>☐ Thrombectomy<br>☐ None

8.18.1	***If 'Endovascular techniques lesion 1' is equal to 'Atherectomy' answer this question:***<br>Atherectomy type lesion 1	◯ Directional atherectomy<br>◯ Rotational atherectomy<br>◯ Orbital atherectomy<br>◯ Laser atherectomy
8.19	Diameter device lesion 1	◯ 2.0 mm<br>◯ 2.5 mm<br>◯ 3.0 mm<br>◯ 3.5 mm<br>◯ 4.0 mm<br>◯ 4.5 mm<br>◯ 5.0 mm<br>◯ 5.5 mm<br>◯ 6.0 mm
8.20	Length device lesion 1<br>*Notice shown if field's value is smaller than 20: 'Are you sure the length is given in mm?'*	______ mm
8.21	Number of inflations device lesion 1	◯ 0<br>◯ 1<br>◯ 2<br>◯ 3<br>◯ 4<br>◯ 5
8.18.2	***If 'Endovascular techniques lesion 1' is equal to 'PTA/stent' answer this question:***<br>Type of stent lesion 1	◯ Bare metal stent (BMS)<br>◯ Covered stent<br>◯ Bioresorbable stent
8.18.3	***If 'Endovascular techniques lesion 1' is equal to 'PTA/stent' answer this question:***<br>Type of stent BE or SE lesion 1	◯ Balloon expandable<br>◯ Self expandable<br>◯ Unknown
8.22	If stenting, show reason for stenting	
8.22.1	***If 'If stenting, show reason for stenting' is equal to '1' answer this question:***<br>Reason stenting lesion 1	◯ Original plan<br>◯ Flow-limiting dissection<br>◯ Significant recoil<br>◯ Study purpose (e.g. trial)<br>◯ Other (please specify)

8.22.2	***If 'If stenting, show reason for stenting' is equal to '1' answer this question:***<br>Number of stents lesion 1	◯ 0<br>◯ 1<br>◯ 2<br>◯ 3<br>◯ 4<br>◯ Unknown
8.22.1.1	***If 'Reason stenting lesion 1' is equal to 'Other (please specify)' answer this question:***<br>Specification reason stenting lesion 1	
8.11.1	***If 'Number of BTK lesions treated' is not equal to '1' answer this question:***<br>Location lesion 2	☐ A. poplitea (P1)<br>☐ A. poplitea (P2)<br>☐ A. poplitea (P3)<br>☐ Truncus tibioperonealis<br>☐ ATA proximal<br>☐ ATA mid<br>☐ ATA distal<br>☐ A. peronea proximal<br>☐ A. peronea mid<br>☐ A. peronea distal<br>☐ ATP proximal<br>☐ ATP mid<br>☐ ATP distal<br>☐ A. dorsalis pedis<br>☐ A. plantaris lateralis<br>☐ A. plantaris medialis<br>☐ Plantar arterial arch
8.11.2	***If 'Number of BTK lesions treated' is not equal to '1' answer this question:***<br>Is lesion 2 continuous POP-crural or crural-pedal?	☐ None<br>☐ POP-crural<br>☐ Crural-pedal
8.11.3	***If 'Number of BTK lesions treated' is not equal to '1' answer this question:***<br>Bifurcation or trifurcation lesion 2?	◯ No<br>◯ Bifurcation lesion<br>◯ Trifurcation lesion
8.11.4	***If 'Number of BTK lesions treated' is not equal to '1' answer this question:***<br>Does lesion 2 consist of multiple stenoses/occlusions?	◯ Yes<br>◯ No
8.11.5	***If 'Number of BTK lesions treated' is not equal to '1' answer this question:***<br>Degree of stenosis based on angiography lesion 2	◯ Stenosis non-significant (<50%)<br>◯ Stenosis significant (≥50%)<br>◯ Total occlusion

8.11.6	***If 'Number of BTK lesions treated' is not equal to '1' answer this question:***<br>Lesion length based on angiography lesion 2	[ ] mm
8.11.2.1	***If 'Is lesion 2 continuous POP-crural or crural-pedal?' is equal to 'POP-crural' answer this question:***<br>Popliteal lesion length lesion 2	[ ] mm
8.11.2.2	***If 'Is lesion 2 continuous POP-crural or crural-pedal?' is equal to 'POP-crural' answer this question:***<br>Popliteal lesion degree of stenosis lesion 2	○ Stenosis non-significant (<50%)<br>○ Stenosis significant (≥50%)<br>○ Total occlusion
8.11.2.3	***If 'Is lesion 2 continuous POP-crural or crural-pedal?' is not equal to 'None' answer this question:***<br>Crural lesion length lesion 2	[ ] mm
8.11.2.4	***If 'Is lesion 2 continuous POP-crural or crural-pedal?' is not equal to 'None' answer this question:***<br>Crural lesion degree of stenosis lesion 2	○ Stenosis non-significant (<50%)<br>○ Stenosis significant (≥50%)<br>○ Total occlusion
8.11.2.5	***If 'Is lesion 2 continuous POP-crural or crural-pedal?' is equal to 'Crural-pedal' answer this question:***<br>Pedal lesion length lesion 2	[ ] mm
8.11.2.6	***If 'Is lesion 2 continuous POP-crural or crural-pedal?' is equal to 'Crural-pedal' answer this question:***<br>Pedal lesion degree of stenosis lesion 2	○ Stenosis non-significant (<50%)<br>○ Stenosis significant (≥50%)<br>○ Total occlusion
8.11.7	***If 'Number of BTK lesions treated' is not equal to '1' answer this question:***<br>Endovascular techniques lesion 2	☐ PTA<br>☐ PTA/stent<br>☐ DCB<br>☐ DES<br>☐ Shockwave balloon (intravascular lithotripsy)<br>☐ Atherectomy<br>☐ Local thrombolysis<br>☐ Thrombectomy<br>☐ None
8.11.7.1	***If 'Endovascular techniques lesion 2' is equal to 'Atherectomy' answer this question:***<br>Atherectomy type lesion 2	○ Directional atherectomy<br>○ Rotational atherectomy<br>○ Orbital atherectomy<br>○ Laser atherectomy

8.11.8	***If 'Number of BTK lesions treated' is not equal to '1' answer this question:***<br>Diameter device lesion 2	◯ 2.0 mm<br>◯ 2.5 mm<br>◯ 3.0 mm<br>◯ 3.5 mm<br>◯ 4.0 mm<br>◯ 4.5 mm<br>◯ 5.0 mm<br>◯ 5.5 mm<br>◯ 6.0 mm
8.11.9	***If 'Number of BTK lesions treated' is not equal to '1' answer this question:***<br>Length device lesion 2<br>*Notice shown if field's value is smaller than 20: 'Are you sure the length is given in mm?'*	[ ] mm
8.11.10	***If 'Number of BTK lesions treated' is not equal to '1' answer this question:***<br>Number of inflations device lesion 2	◯ 0<br>◯ 1<br>◯ 2<br>◯ 3<br>◯ 4<br>◯ 5
8.11.7.2	***If 'Endovascular techniques lesion 2' is equal to 'PTA/stent' answer this question:***<br>Type of stent lesion 2	◯ Bare metal stent (BMS)<br>◯ Covered stent<br>◯ Bioresorbable stent
8.11.7.3	***If 'Endovascular techniques lesion 2' is equal to 'PTA/stent' answer this question:***<br>Type of stent BE or SE lesion 2	◯ Balloon expandable<br>◯ Self expandable<br>◯ Unknown
8.23	If stenting, show reason for stenting2	
8.23.1	***If 'If stenting, show reason for stenting2' is equal to '1' answer this question:***<br>Reason stenting lesion 2	◯ Original plan<br>◯ Flow-limiting dissection<br>◯ Significant recoil<br>◯ Study purpose (e.g. trial)<br>◯ Other (please specify)
8.23.2	***If 'If stenting, show reason for stenting2' is equal to '1' answer this question:***<br>Number of stents lesion 2	◯ 0<br>◯ 1<br>◯ 2<br>◯ 3<br>◯ 4<br>◯ Unknown

8.23.1.1	***If 'Reason stenting lesion 2' is equal to 'Other (please specify)' answer this question:***<br>Specification reason stenting lesion 2	
8.24	If 3-4 BTK lesions revascularized, show info lesion 3	
8.24.1	***If 'If 3-4 BTK lesions revascularized, show info lesion 3' is equal to '1' answer this question:***<br>Location lesion 3	☐ A. poplitea (P1)<br>☐ A. poplitea (P2)<br>☐ A. poplitea (P3)<br>☐ Truncus tibioperonealis<br>☐ ATA proximal<br>☐ ATA mid<br>☐ ATA distal<br>☐ A. peronea proximal<br>☐ A. peronea mid<br>☐ A. peronea distal<br>☐ ATP proximal<br>☐ ATP mid<br>☐ ATP distal<br>☐ A. dorsalis pedis<br>☐ A. plantaris lateralis<br>☐ A. plantaris medialis<br>☐ Plantar arterial arch
8.24.2	***If 'If 3-4 BTK lesions revascularized, show info lesion 3' is equal to '1' answer this question:***<br>Is lesion 3 continuous POP-crural or crural-pedal?	☐ None<br>☐ POP-crural<br>☐ Crural-pedal
8.24.3	***If 'If 3-4 BTK lesions revascularized, show info lesion 3' is equal to '1' answer this question:***<br>Does lesion 3 consist of multiple stenoses/occlusions?	◯ Yes<br>◯ No
8.24.4	***If 'If 3-4 BTK lesions revascularized, show info lesion 3' is equal to '1' answer this question:***<br>Degree of stenosis based on angiography lesion 3	◯ Stenosis non-significant (<50%)<br>◯ Stenosis significant (≥50%)<br>◯ Total occlusion
8.24.5	***If 'If 3-4 BTK lesions revascularized, show info lesion 3' is equal to '1' answer this question:***<br>Lesion length based on angiography lesion 3	______ mm
8.24.2.1	***If 'Is lesion 3 continuous POP-crural or crural-pedal?' is equal to 'POP-crural' answer this question:***<br>Popliteal lesion length lesion 3	______ mm
8.24.2.2	***If 'Is lesion 3 continuous POP-crural or crural-pedal?' is equal to 'POP-crural' answer this question:***<br>Popliteal lesion degree of stenosis lesion 3	◯ Stenosis non-significant (<50%)<br>◯ Stenosis significant (≥50%)<br>◯ Total occlusion

8.24.2.3	***If 'Is lesion 3 continuous POP-crural or crural-pedal?' is not equal to 'None' answer this question:***<br>Crural lesion length lesion 3	[ ] mm
8.24.2.4	***If 'Is lesion 3 continuous POP-crural or crural-pedal?' is not equal to 'None' answer this question:***<br>Crural lesion degree of stenosis lesion 3	○ Stenosis non-significant (<50%)<br>○ Stenosis significant (≥50%)<br>○ Total occlusion
8.24.2.5	***If 'Is lesion 3 continuous POP-crural or crural-pedal?' is equal to 'Crural-pedal' answer this question:***<br>Pedal lesion length lesion 3	[ ] mm
8.24.2.6	***If 'Is lesion 3 continuous POP-crural or crural-pedal?' is equal to 'Crural-pedal' answer this question:***<br>Pedal lesion degree of stenosis lesion 3	○ Stenosis non-significant (<50%)<br>○ Stenosis significant (≥50%)<br>○ Total occlusion
8.24.6	***If 'If 3-4 BTK lesions revascularized, show info lesion 3' is equal to '1' answer this question:***<br>Endovascular techniques lesion 3	☐ PTA<br>☐ PTA/stent<br>☐ DCB<br>☐ DES<br>☐ Shockwave balloon (intravascular lithotripsy)<br>☐ Atherectomy<br>☐ Local thrombolysis<br>☐ Thrombectomy<br>☐ None
8.24.6.1	***If 'Endovascular techniques lesion 3' is equal to 'Atherectomy' answer this question:***<br>Atherectomy type lesion 3	○ Directional atherectomy<br>○ Rotational atherectomy<br>○ Orbital atherectomy<br>○ Laser atherectomy
8.24.7	***If 'If 3-4 BTK lesions revascularized, show info lesion 3' is equal to '1' answer this question:***<br>Diameter device lesion 3	○ 2.0 mm<br>○ 2.5 mm<br>○ 3.0 mm<br>○ 3.5 mm<br>○ 4.0 mm<br>○ 4.5 mm<br>○ 5.0 mm<br>○ 5.5 mm<br>○ 6.0 mm
8.24.8	***If 'If 3-4 BTK lesions revascularized, show info lesion 3' is equal to '1' answer this question:***<br>Length device lesion 3<br>*Notice shown if field's value is smaller than 20: 'Are you sure the length is given in mm?'*	[ ] mm

8.24.9	***If 'If 3-4 BTK lesions revascularized, show info lesion 3' is equal to '1' answer this question:***<br>Number of inflations device lesion 3	◯ 0<br>◯ 1<br>◯ 2<br>◯ 3<br>◯ 4<br>◯ 5
8.24.6.2	***If 'Endovascular techniques lesion 3' is equal to 'PTA/stent' answer this question:***<br>Type of stent lesion 3	◯ Bare metal stent (BMS)<br>◯ Covered stent<br>◯ Bioresorbable stent
8.24.6.3	***If 'Endovascular techniques lesion 3' is equal to 'PTA/stent' answer this question:***<br>Type of stent BE or SE lesion 3	◯ Balloon expandable<br>◯ Self expandable<br>◯ Unknown
8.25	If stenting, show reason for stenting3	
8.25.1	***If 'If stenting, show reason for stenting3' is equal to '1' answer this question:***<br>Reason stenting lesion 3	◯ Original plan<br>◯ Flow-limiting dissection<br>◯ Significant recoil<br>◯ Study purpose (e.g. trial)<br>◯ Other (please specify)
8.25.2	***If 'If stenting, show reason for stenting3' is equal to '1' answer this question:***<br>Number of stents lesion 3	◯ 0<br>◯ 1<br>◯ 2<br>◯ 3<br>◯ 4<br>◯ Unknown
8.25.1.1	***If 'Reason stenting lesion 3' is equal to 'Other (please specify)' answer this question:***<br>Specification reason stenting lesion 3	

8.11.11	***If 'Number of BTK lesions treated' is equal to '4' answer this question:***<br>Location lesion 4	☐ A. poplitea (P1)<br>☐ A. poplitea (P2)<br>☐ A. poplitea (P3)<br>☐ Truncus tibioperonealis<br>☐ ATA proximal<br>☐ ATA mid<br>☐ ATA distal<br>☐ A. peronea proximal<br>☐ A. peronea mid<br>☐ A. peronea distal<br>☐ ATP proximal<br>☐ ATP mid<br>☐ ATP distal<br>☐ A. dorsalis pedis<br>☐ A. plantaris lateralis<br>☐ A. plantaris medialis<br>☐ Plantar arterial arch
8.11.12	***If 'Number of BTK lesions treated' is equal to '4' answer this question:***<br>Is lesion 4 continuous POP-crural or crural-pedal?	☐ None<br>☐ POP-crural<br>☐ Crural-pedal
8.11.13	***If 'Number of BTK lesions treated' is equal to '4' answer this question:***<br>Does lesion 4 consist of multiple stenoses/occlusions?	○ Yes<br>○ No
8.11.14	***If 'Number of BTK lesions treated' is equal to '4' answer this question:***<br>Degree of stenosis based on angiography lesion 4	○ Stenosis non-significant (<50%)<br>○ Stenosis significant (≥50%)<br>○ Total occlusion
8.11.15	***If 'Number of BTK lesions treated' is equal to '4' answer this question:***<br>Lesion length based on angiography lesion 4	______ mm
8.11.12.1	***If 'Is lesion 4 continuous POP-crural or crural-pedal?' is equal to 'POP-crural' answer this question:***<br>Popliteal lesion length lesion 4	______ mm
8.11.12.2	***If 'Is lesion 4 continuous POP-crural or crural-pedal?' is equal to 'POP-crural' answer this question:***<br>Popliteal lesion degree of stenosis lesion 4	○ Stenosis non-significant (<50%)<br>○ Stenosis significant (≥50%)<br>○ Total occlusion
8.11.12.3	***If 'Is lesion 4 continuous POP-crural or crural-pedal?' is not equal to 'None' answer this question:***<br>Crural lesion length lesion 4	______ mm

8.11.12.4	***If 'Is lesion 4 continuous POP-crural or crural-pedal?' is not equal to 'None' answer this question:***<br>Crural lesion degree of stenosis lesion 4	◯ Stenosis non-significant (<50%)<br>◯ Stenosis significant (≥50%)<br>◯ Total occlusion
8.11.12.5	***If 'Is lesion 4 continuous POP-crural or crural-pedal?' is equal to 'Crural-pedal' answer this question:***<br>Pedal lesion length lesion 4	[ ] mm
8.11.12.6	***If 'Is lesion 4 continuous POP-crural or crural-pedal?' is equal to 'Crural-pedal' answer this question:***<br>Pedal lesion degree of stenosis lesion 4	◯ Stenosis non-significant (<50%)<br>◯ Stenosis significant (≥50%)<br>◯ Total occlusion
8.11.16	***If 'Number of BTK lesions treated' is equal to '4' answer this question:***<br>Endovascular techniques lesion 4	☐ PTA<br>☐ PTA/stent<br>☐ DCB<br>☐ DES<br>☐ Shockwave balloon (intravascular lithotripsy)<br>☐ Atherectomy<br>☐ Local thrombolysis<br>☐ Thrombectomy<br>☐ None
8.11.16.1	***If 'Endovascular techniques lesion 4' is equal to 'Atherectomy' answer this question:***<br>Atherectomy type lesion 4	◯ Directional atherectomy<br>◯ Rotational atherectomy<br>◯ Orbital atherectomy<br>◯ Laser atherectomy
8.11.17	***If 'Number of BTK lesions treated' is equal to '4' answer this question:***<br>Diameter device lesion 4	◯ 2.0 mm<br>◯ 2.5 mm<br>◯ 3.0 mm<br>◯ 3.5 mm<br>◯ 4.0 mm<br>◯ 4.5 mm<br>◯ 5.0 mm<br>◯ 5.5 mm<br>◯ 6.0 mm
8.11.18	***If 'Number of BTK lesions treated' is equal to '4' answer this question:***<br>Length device lesion 4<br>*Notice shown if field's value is smaller than 20: 'Are you sure the length is given in mm?'*	[ ] mm

8.11.19	***If 'Number of BTK lesions treated' is equal to '4' answer this question:***<br>Number of inflations device lesion 4	◯ 0<br>◯ 1<br>◯ 2<br>◯ 3<br>◯ 4<br>◯ 5
8.11.16.2	***If 'Endovascular techniques lesion 4' is equal to 'PTA/stent' answer this question:***<br>Type of stent lesion 4	◯ Bare metal stent (BMS)<br>◯ Covered stent<br>◯ Bioresorbable stent
8.11.16.3	***If 'Endovascular techniques lesion 4' is equal to 'PTA/stent' answer this question:***<br>Type of stent BE or SE lesion 4	◯ Balloon expandable<br>◯ Self expandable<br>◯ Unknown
8.26	If stenting, show reason for stenting4	
8.26.1	***If 'If stenting, show reason for stenting4' is equal to '1' answer this question:***<br>Reason stenting lesion 4	◯ Original plan<br>◯ Flow-limiting dissection<br>◯ Significant recoil<br>◯ Study purpose (e.g. trial)<br>◯ Other (please specify)
8.26.2	***If 'If stenting, show reason for stenting4' is equal to '1' answer this question:***<br>Number of stents lesion 4	◯ 0<br>◯ 1<br>◯ 2<br>◯ 3<br>◯ 4<br>◯ Unknown
8.26.1.1	***If 'Reason stenting lesion 4' is equal to 'Other (please specify)' answer this question:***<br>Specification reason stenting lesion 4	
8.27	Full specification endovascular intervention performed	

9. Intervention - Simultaneous interventions

Number	Question	Answers

9.1	Simultaneous interventions same limb	☐ Endovascular<br>☐ Femoral endarterectomy<br>☐ Bypass<br>☐ Limflow procedure<br>☐ None
9.1.1	***If 'Simultaneous interventions same limb' is equal to 'Bypass' answer this question:***<br>Type of bypass surgery same limb	☐ Aortofemoral bypass<br>☐ Axillofemoral bypass<br>☐ Fempop supragenual bypass<br>☐ Fempop infragenual bypass<br>☐ Femcrural bypass<br>☐ Femfem crossover bypass<br>☐ Other
9.1.2	***If 'Simultaneous interventions same limb' is equal to 'Bypass' answer this question:***<br>Specification type of bypass surgery same limb text	
9.1.3	***If 'Simultaneous interventions same limb' is equal to 'Bypass' answer this question:***<br>Material bypass same limb	○ Autologous<br>○ PTFE (Polytetrafluoroethylene)<br>○ Dacron (not heparin bonded)<br>○ HUV (Human umbilical vein)<br>○ HBD (Heparin bonded dacron)
9.1.4	***If 'Simultaneous interventions same limb' is equal to 'Endovascular' answer this question:***<br>Location other endovascular interventions same limb	☐ Aorta<br>☐ AIC<br>☐ AIE<br>☐ AFC<br>☐ AFS<br>☐ AFP<br>☐ A. poplitea
9.1.5	***If 'Simultaneous interventions same limb' is equal to 'Endovascular' answer this question:***<br>Number of ATK lesions treated	○ 1<br>○ 2<br>○ 3<br>○ 4

9.1.6	***If 'Simultaneous interventions same limb' is equal to 'Endovascular' answer this question:*** Type of devices used in ATK lesions same limb	☐ PTA<br>☐ PTA/stent<br>☐ DCB<br>☐ DES<br>☐ Shockwave balloon (intravascular lithotripsy)<br>☐ Atherectomy<br>☐ Local thrombolysis<br>☐ Thrombectomy<br>☐ None
9.1.7	***If 'Simultaneous interventions same limb' is equal to 'Endovascular' answer this question:*** Specification ATK endovascular interventions same limb	
9.2	Simultaneous interventions other limb	☐ Endovascular<br>☐ Femoral endarterectomy<br>☐ Bypass<br>☐ Limflow procedure<br>☐ None
9.2.1	***If 'Simultaneous interventions other limb' is equal to 'Bypass' answer this question:*** Type of bypass surgery other limb	☐ Aortofemoral bypass<br>☐ Axillofemoral bypass<br>☐ Fempop supragenual bypass<br>☐ Fempop infragenual bypass<br>☐ Femcrural bypass<br>☐ Femfem crossover bypass<br>☐ Other
9.2.2	***If 'Simultaneous interventions other limb' is equal to 'Bypass' answer this question:*** Specification type of bypass surgery other limb	

9.2.3	***If 'Simultaneous interventions other limb' is equal to 'Endovascular' answer this question:***<br>Location endovascular interventions other limb	☐ Aorta<br>☐ AIC<br>☐ AIE<br>☐ AFC<br>☐ AFS<br>☐ A. poplitea (P1)<br>☐ A. poplitea (P2-P3)<br>☐ ATA<br>☐ Truncus tibioperonealis<br>☐ A. peroneale<br>☐ ATP<br>☐ Pedal arteries
9.2.4	***If 'Simultaneous interventions other limb' is equal to 'Endovascular' answer this question:***<br>Number of lesions treated other limb	○ 1<br>○ 2<br>○ 3<br>○ 4
9.2.5	***If 'Simultaneous interventions other limb' is equal to 'Endovascular' answer this question:***<br>Type of devices used in other limb	☐ PTA<br>☐ PTA/stent<br>☐ DCB<br>☐ DES<br>☐ Shockwave balloon (intravascular lithotripsy)<br>☐ Atherectomy<br>☐ Local thrombolysis<br>☐ Thrombectomy<br>☐ None
9.2.6	***If 'Simultaneous interventions other limb' is equal to 'Endovascular' answer this question:***<br>Specification simultaneous endovascular interventions other limb	
9.3	Simultaneous amputations or wound debridement performed same leg	☐ None<br>☐ Only wound debridement<br>☐ MTP-amputation<br>☐ TMT-amputation<br>☐ Forefoot amputation<br>☐ Ankle amputation<br>☐ Other limb

9.3.1	***If 'Simultaneous amputations or wound debridement performed same leg' is equal to 'MTP-amputation' answer this question:***<br>Digits simultaneously amputated MTP	☐ Dig 1<br>☐ Dig 2<br>☐ Dig 3<br>☐ Dig 4<br>☐ Dig 5
9.3.2	***If 'Simultaneous amputations or wound debridement performed same leg' is equal to 'TMT-amputation' answer this question:***<br>Digits simultaneously amputated TMT	☐ Dig 1<br>☐ Dig 2<br>☐ Dig 3<br>☐ Dig 4<br>☐ Dig 5
9.3.3	***If 'Simultaneous amputations or wound debridement performed same leg' is equal to 'Other limb' answer this question:***<br>Simultaneous amputations performed other leg	☐ Dig 1<br>☐ Dig 2<br>☐ Dig 3<br>☐ Dig 4<br>☐ Dig 5<br>☐ Forefoot<br>☐ Ankle<br>☐ Below the knee<br>☐ Through the knee<br>☐ Above the knee

10. Intervention - Peroperative complications

Number	Question	Answers
10.1	Was procedural success accomplished in lesion 1?	○ Yes<br>○ No
10.1.1	***If 'Was procedural success accomplished in lesion 1?' is equal to 'No' answer this question:***<br>Reason no procedural success lesion 1	○ Unsuccessful recanalization<br>○ Persistent stenosis > 30%<br>○ Other
10.1.1.1	***If 'Reason no procedural success lesion 1' is equal to 'Unsuccessful recanalization' answer this question:***<br>Types of recanalization attempted lesion 1	○ Antegrade<br>○ Retrograde<br>○ Both antegrade and retrograde
10.1.1.2	***If 'Reason no procedural success lesion 1' is equal to 'Other' answer this question:***<br>Specification reason no proc success lesion 1	

10.2	***If 'Number of BTK lesions treated' is not equal to '1' answer this question:***<br>Was procedural success accomplished in lesion 2?	◯ Yes<br>◯ No
10.2.1	***If 'Was procedural success accomplished in lesion 2?' is equal to 'No' answer this question:***<br>Reason no procedural success lesion 2	◯ Unsuccessful recanalization<br>◯ Persistent stenosis > 30%<br>◯ Other
10.2.1.1	***If 'Reason no procedural success lesion 2' is equal to 'Unsuccessful recanalization' answer this question:***<br>Types of recanalization attempted lesion 2	◯ Antegrade<br>◯ Retrograde<br>◯ Both antegrade and retrograde
10.2.1.2	***If 'Reason no procedural success lesion 2' is equal to 'Other' answer this question:***<br>Specification reason no proc success lesion 2	
10.3	***If 'If 3-4 BTK lesions revascularized, show info lesion 3' is equal to '1' answer this question:***<br>Was procedural success accomplished in lesion 3?	◯ Yes<br>◯ No
10.3.1	***If 'Was procedural success accomplished in lesion 3?' is equal to 'No' answer this question:***<br>Reason no procedural success lesion 3	◯ Unsuccessful recanalization<br>◯ Persistent stenosis > 30%<br>◯ Other
10.3.1.1	***If 'Reason no procedural success lesion 3' is equal to 'Unsuccessful recanalization' answer this question:***<br>Types of recanalization attempted lesion 3	◯ Antegrade<br>◯ Retrograde<br>◯ Both antegrade and retrograde
10.3.1.2	***If 'Reason no procedural success lesion 3' is equal to 'Other' answer this question:***<br>Specification reason no proc success lesion 3	
10.4	***If 'Number of BTK lesions treated' is equal to '4' answer this question:***<br>Was procedural success accomplished in lesion 4?	◯ Yes<br>◯ No
10.4.1	***If 'Was procedural success accomplished in lesion 4?' is equal to 'No' answer this question:***<br>Reason no procedural success lesion 4	◯ Unsuccessful recanalization<br>◯ Persistent stenosis > 30%<br>◯ Other
10.4.1.1	***If 'Reason no procedural success lesion 4' is equal to 'Unsuccessful recanalization' answer this question:***<br>Types of recanalization attempted lesion 4	◯ Antegrade<br>◯ Retrograde<br>◯ Both antegrade and retrograde

10.4.1.2	***If 'Reason no procedural success lesion 4' is equal to 'Other' answer this question:***<br>Specification reason no proc success lesion 4	
10.5	Periprocedural vascular complications	☐ None<br>☐ Acute occlusion<br>☐ Distal embolization<br>☐ Perforation<br>☐ Device failure<br>☐ Other (please specify)
10.5.1	***If 'Periprocedural vascular complications' is not equal to 'None' answer this question:***<br>Specification periprocedural vascular complications	
10.6	Periprocedural general complications<br>*Notice shown if field's value is not equal to None: 'Please also fill in the report form ''adverse events''.'*	☐ None<br>☐ Acute myocardial infarction<br>☐ Cerebrovascular accident<br>☐ Death
10.6.1	***If 'Periprocedural general complications' is equal to 'Death' answer this question:***<br>Cause of periprocedural death	○ Myocardial infarction/cardiac arrest<br>○ Astma cardiale<br>○ Cerebrovascular accident (ischaemic)<br>○ Cerebrovascular accident (bleeding)<br>○ Sepsis<br>○ Acute kidney failure<br>○ Pulmonary embolism<br>○ Hypovolemic shock<br>○ Anaphylactic shock (e.g. allergic reaction)<br>○ Cancer<br>○ Unknown<br>○ Other
10.6.2	***If 'Periprocedural general complications' is equal to 'Death' answer this question:***<br>Specification cause of periprocedural death	

11. Intervention - GLASS (based on angiography)

Number	Question	Answers

11.1	Outflow vessels present before intervention	◯ 0<br>◯ 1<br>◯ 2<br>◯ 3
11.2	Outflow vessels present after intervention	◯ 0<br>◯ 1<br>◯ 2<br>◯ 3
11.3	Number of lesions to fill in TASC/GLASS crural	◯ 1<br>◯ 2<br>◯ 3
11.4	TASC classification fempop figure	
11.5	TASC classification femoropopliteal	◯ 0<br>◯ A<br>◯ B<br>◯ C<br>◯ D
11.6	TASC classification crural figure	
11.7	TASC classification crural 1	◯ 0<br>◯ A<br>◯ B<br>◯ C<br>◯ D
11.3.1	***If 'Number of lesions to fill in TASC/GLASS crural' is not equal to '1' answer this question:***<br>TASC classification crural 2	◯ 0<br>◯ A<br>◯ B<br>◯ C<br>◯ D
11.3.2	***If 'Number of lesions to fill in TASC/GLASS crural' is equal to '3' answer this question:***<br>TASC classification crural 3	◯ 0<br>◯ A<br>◯ B<br>◯ C<br>◯ D
11.8	GLASS femoropopliteal stage figure	

11.9	GLASS femoropopliteal stage treated lesion	◯ 0<br>◯ 1<br>◯ 2<br>◯ 3<br>◯ 4
11.10	GLASS femoropopliteal stage total fempop	◯ 0<br>◯ 1<br>◯ 2<br>◯ 3<br>◯ 4
11.11	Femoropopliteal calcification	◯ Severe calcification<br>◯ Moderate or low calcification
11.12	GLASS infrapopliteal stage figure	
11.13	GLASS infrapopliteal stage 1	◯ 0<br>◯ 1<br>◯ 2<br>◯ 3<br>◯ 4
11.3.3	***If 'Number of lesions to fill in TASC/GLASS crural' is not equal to '1' answer this question:***<br>GLASS infrapopliteal stage 2	◯ 0<br>◯ 1<br>◯ 2<br>◯ 3<br>◯ 4
11.3.4	***If 'Number of lesions to fill in TASC/GLASS crural' is equal to '3' answer this question:***<br>GLASS infrapopliteal stage 3	◯ 0<br>◯ 1<br>◯ 2<br>◯ 3<br>◯ 4
11.14	GLASS infrapopliteal stage of best crural artery	◯ 0<br>◯ 1<br>◯ 2<br>◯ 3<br>◯ 4
11.15	Infrapopliteal calcification 1	◯ Severe calcification<br>◯ Moderate or low calcification

11.3.5	***If 'Number of lesions to fill in TASC/GLASS crural' is not equal to '1' answer this question:***<br>Infrapopliteal calcification 2	◯ Severe calcification<br>◯ Moderate or low calcification
11.3.6	***If 'Number of lesions to fill in TASC/GLASS crural' is equal to '3' answer this question:***<br>Infrapopliteal calcification 3	◯ Severe calcification<br>◯ Moderate or low calcification
11.16	GLASS Inframalleolar stage figure	
11.17	GLASS Inframalleolar stage	◯ 0<br>◯ 1<br>◯ 2
11.18	***If 'Imaging modalities available' is equal to 'CTA' answer this question:***<br>PACSS classification figure	
11.19	***If 'Imaging modalities available' is equal to 'CTA' answer this question:***<br>PACSS classification lesion 1	◯ 0<br>◯ 1<br>◯ 2<br>◯ 3<br>◯ 4
11.20	***If 'Imaging modalities available' is equal to 'CTA' answer this question:***<br>Calcification 3 categories lesion 1	◯ Minimal calcification<br>◯ Intermediate calcification<br>◯ Extensive calcification
11.21	If CTA and 2 lesions thans show calcification lesion	
11.21.1	***If 'If CTA and 2 lesions thans show calcification lesion' is equal to '1' answer this question:***<br>PACSS classification lesion 2	◯ 0<br>◯ 1<br>◯ 2<br>◯ 3<br>◯ 4
11.21.2	***If 'If CTA and 2 lesions thans show calcification lesion' is equal to '1' answer this question:***<br>Calcification 3 categories lesion 2	◯ Minimal calcification<br>◯ Intermediate calcification<br>◯ Extensive calcification
11.22	If CTA and 3 lesions thans show calcification lesion	
11.22.1	***If 'If CTA and 3 lesions thans show calcification lesion' is equal to '1' answer this question:***<br>PACSS classification lesion 3	◯ 0<br>◯ 1<br>◯ 2<br>◯ 3<br>◯ 4

11.22.2	***If 'If CTA and 3 lesions thans show calcification lesion' is equal to '1' answer this question:***<br>Calcification 3 categories lesion 3	○ Minimal calcification<br>○ Intermediate calcification<br>○ Extensive calcification
11.23	If CTA and 4 lesions thans show calcification lesion	
11.23.1	***If 'If CTA and 4 lesions thans show calcification lesion' is equal to '1' answer this question:***<br>PACSS classification lesion 4	○ 0<br>○ 1<br>○ 2<br>○ 3<br>○ 4
11.23.2	***If 'If CTA and 4 lesions thans show calcification lesion' is equal to '1' answer this question:***<br>Calcification 3 categories lesion 4	○ Minimal calcification<br>○ Intermediate calcification<br>○ Extensive calcification

12. Postoperative phase (4 weeks) - Ankle-brachial-index

Number	Question	Answers
12.1	Date ABI postinterventional	[ ] [ ] [ ] *(dd-mm-yyyy)*
12.2	Blood pressure arm postinterventional	[ ]
12.3	Arteries ankle in which ankle pressure is measured	☐ ATP<br>☐ ADP<br>☐ Unknown
12.3.1	***If 'Arteries ankle in which ankle pressure is measured' is equal to 'ATP' answer this question:***<br>Blood pressure ATP postinterventional	[ ]
12.3.1.1	***If 'Blood pressure ATP postinterventional' is greater or equal than '1' answer this question:***<br>ABI based on ATP postinterventional	
12.3.2	***If 'Arteries ankle in which ankle pressure is measured' is equal to 'ADP' answer this question:***<br>Blood pressure ADP postinterventional	[ ]
12.3.2.1	***If 'Blood pressure ADP postinterventional' is greater or equal than '1' answer this question:***<br>ABI based on ADP postinterventional	
12.4	Highest blood pressure in ankle postinterventional	[ ]
12.4.1	***If 'Highest blood pressure in ankle postinterventional' is greater or equal than '1' answer this question:***<br>Highest ABI in ankle postinterventional	

12.4.2	***If 'Highest blood pressure in ankle postinterventional' is smaller than '5' answer this question:***<br>Highest ABI fill in yourself	
12.4.3	***If 'Highest blood pressure in ankle postinterventional' is smaller than '5' answer this question:***<br>Reason no ankle pressure postinterventional	○ Not compressible<br>○ Not measured<br>○ Other
12.4.3.1	***If 'Reason no ankle pressure postinterventional' is equal to 'Other' answer this question:***<br>Specification reason no ankle pressure	
12.5	Toe pressure postinterventional	
12.5.1	***If 'Toe pressure postinterventional' is greater or equal than '1' answer this question:***<br>TBI postinterventional	
12.5.2	***If 'Toe pressure postinterventional' is greater than '-1' answer this question:***<br>Toe pressure postinterventional curve	
12.5.3	***If 'Toe pressure postinterventional' is greater than '-1' answer this question:***<br>Toe pressure acceleration time	
12.6	***If 'Toe pressure' is greater than '-1' answer this question:***<br>Ankle pressure curve (optional)	
12.7	***If 'Toe pressure' is greater than '-1' answer this question:***<br>Ankle (ATP) pressure acceleration time	
12.8	***If 'Toe pressure' is greater than '-1' answer this question:***<br>Ankle (ADP) pressure acceleration time	

13. Postoperative phase (4 weeks) - Hospital stay

Number	Question	Answers
13.1	Date of discharge	*(dd-mm-yyyy)*
13.2	Days in hospital after intervention	

13.3	Postprocedural vascular complications<br>*Notice shown if field's value is equal to Acute occlusion: 'Please also fill in the report form ''adverse events''.'*	☐ None<br>☐ Acute occlusion<br>☐ Distal embolization<br>☐ Perforation<br>☐ Severe bleeding (for which re-intervention required)<br>☐ Pseudoaneurysm<br>☐ Surgical site infection<br>☐ Compartment syndrome
13.4	Postprocedural general complications<br>*Notice shown if field's value is equal to Acute myocardial infarction: 'Please also fill in the report form ''adverse events''.'*<br>*Notice shown if field's value is equal to Stroke: 'Please also fill in the report form ''adverse events''.'*<br>*Notice shown if field's value is equal to Death: 'Please also fill in the report form ''adverse events''.'*	☐ None<br>☐ Acute myocardial infarction<br>☐ Stroke<br>☐ Contrast-induced nephropathy<br>☐ Death
13.4.1	***If 'Postprocedural general complications' is equal to 'Contrast-induced nephropathy' answer this question:***<br>Creatinine level postprocedural	______ umol/L
13.5	MDRD GFR level postprocedural	
13.4.2	***If 'Postprocedural general complications' is equal to 'Death' answer this question:***<br>Cause of postprocedural death	○ Myocardial infarction/cardiac arrest<br>○ Astma cardiale<br>○ Cerebrovascular accident (ischaemic)<br>○ Cerebrovascular accident (bleeding)<br>○ Sepsis<br>○ Acute kidney failure<br>○ Pulmonary embolism<br>○ Hypovolemic shock<br>○ Anaphylactic shock (e.g. allergic reaction)<br>○ Cancer<br>○ Unknown<br>○ Other
13.4.3	***If 'Postprocedural general complications' is equal to 'Death' answer this question:***<br>Specification cause of postprocedural death	

13.6	Re-intervention postprocedural<br>*Notice shown if field's value is not equal to None: 'Please also fill in the report form "adverse events" if any form of revascularization or amputation was performed.'*	☐ None<br>☐ Endovascular revascularization<br>☐ Bypass surgery<br>☐ Minor amputation(s)<br>☐ Major amputation<br>☐ Evacuation of hematoma<br>☐ Thrombin injections<br>☐ Local thrombolysis<br>☐ Systemic thrombolysis<br>☐ Thrombectomy/thrombosuction<br>☐ Fasciotomy<br>☐ Other (please specify)
13.6.1	***If 'Re-intervention postprocedural' is not equal to 'None' answer this question:***<br>Specification re-intervention postprocedural	
13.7	Antithrombotics at discharge	☐ None<br>☐ Acetylsalicylzuur (or carbasalaatcalcium)<br>☐ P2Y12-inhibitor (e.g. clopidogrel, ticagrelor, prasugrel)<br>☐ Dipyridamol<br>☐ Vitamin K antagonist<br>☐ DOAC (e.g. rivaroxaban, dabigatran)<br>☐ Heparins
13.8	If patient uses DAPT, show for how long	
13.8.1	***If 'If patient uses DAPT, show for how long' is equal to '1' answer this question:***<br>If DAPT, for which time interval?	○ 1 month<br>○ 3 months<br>○ 6 months<br>○ 12 months<br>○ Lifelong<br>○ Unknown<br>○ Other (please specify)<br>○ No DAPT
13.8.1.1	***If 'If DAPT, for which time interval?' is equal to 'Other (please specify)' answer this question:***<br>Specification length of DAPT	
13.9	If patient uses ascal and VKA, show for how long	

13.9.1	***If 'If patient uses ascal and VKA, show for how long' is equal to '1' answer this question:***<br>If ascal and VKA, for which time interval?	◯ 1 month<br>◯ 3 months<br>◯ 6 months<br>◯ 12 months<br>◯ Lifelong<br>◯ Unknown<br>◯ Other (please specify)<br>◯ No DAPT
13.9.1.1	***If 'If ascal and VKA, for which time interval?' is equal to 'Other (please specify)' answer this question:***<br>Specification length of ascal and VKA	
13.10	If patient uses P2Y12 inhibitor and VKA, show for how long	
13.10.1	***If 'If patient uses P2Y12 inhibitor and VKA, show for how long' is equal to '1' answer this question:***<br>If P2Y12 inhibitor and VKA, for which time interval?	◯ 1 month<br>◯ 3 months<br>◯ 6 months<br>◯ 12 months<br>◯ Lifelong<br>◯ Unknown<br>◯ Other (please specify)<br>◯ No DAPT
13.10.1.1	***If 'If P2Y12 inhibitor and VKA, for which time interval?' is equal to 'Other (please specify)' answer this question:***<br>Specification length of P2Y12 inhibitor and VKA	
13.11	If patient uses ascal and DOAC, show for how long	
13.11.1	***If 'If patient uses ascal and DOAC, show for how long' is equal to '1' answer this question:***<br>If ascal and DOAC, for which time interval?	◯ 1 month<br>◯ 3 months<br>◯ 6 months<br>◯ 12 months<br>◯ Lifelong<br>◯ Unknown<br>◯ Other (please specify)<br>◯ No DAPT
13.11.1.1	***If 'If ascal and DOAC, for which time interval?' is equal to 'Other (please specify)' answer this question:***<br>Specification length of ascal and DOAC	

13.11.2	***If 'If patient uses ascal and DOAC, show for how long' is equal to '1' answer this question:***<br>Is the patient using rivaroxaban 2dd 2.5mg + ascal?	◯ Yes<br>◯ No
13.12	If patient uses P2Y12 inhibitor and DOAC, show for how long	
13.12.1	***If 'If patient uses P2Y12 inhibitor and DOAC, show for how long' is equal to '1' answer this question:***<br>If P2Y12 inhibitor and DOAC, for which time interval?	◯ 1 month<br>◯ 3 months<br>◯ 6 months<br>◯ 12 months<br>◯ Lifelong<br>◯ Unknown<br>◯ Other (please specify)<br>◯ No DAPT
13.12.1.1	***If 'If P2Y12 inhibitor and DOAC, for which time interval?' is equal to 'Other (please specify)' answer this question:***<br>Specification length of P2Y12 inhibitor and DOAC	
13.13	Specification (and indication) antithrombotics postoperative	
13.14	***If 'Was procedural success accomplished in lesion 1?' is equal to 'No' answer this question:***<br>After failed attempt of revascularization, is this a case of desert foot?	◯ Yes<br>◯ No, because other crural lesions have been successfully revascularized.<br>◯ No, because there is still blood flow in the foot despite no lesions have been revascularized.
13.14.1	***If 'After failed attempt of revascularization, is this a case of desert foot?' is not equal to 'No, because other crural lesions have been successfully revascularized.' answer this question:***<br>After failed attempt of revascularization, what is the new plan?	☐ New attempt of endovascular revascularization<br>☐ Open revasularization<br>☐ Major amputation<br>☐ Hyperbaric oxygen therapy<br>☐ Prostanoids (e.g. iloprost)<br>☐ Wait and see<br>☐ Other (please specify)
13.14.2	***If 'After failed attempt of revascularization, is this a case of desert foot?' is not equal to 'No, because other crural lesions have been successfully revascularized.' answer this question:***<br>Specification new plan after failed attempt of revascularization	

14. Postoperative phase (4 weeks) - Limb staging (WIfI)

Number	Question	Answers
14.1	***If 'If CLTI, than...' is equal to '1' answer this question:***<br>Wound (WIfI) at discharge	◯ 0: No ulcer and no gangrene<br>◯ 1: Small ulcer and no gangrene<br>◯ 2: Deep ulcer or gangrene limited to toes<br>◯ 3: Extensive ulcer or extensive gangrene
14.2	***If 'If CLTI, than...' is equal to '1' answer this question:***<br>Ischemia (WIfI) baseline based on...	
14.3	***If 'If CLTI, than...' is equal to '1' answer this question:***<br>Ischemia (WIfI) at discharge	◯ 0: > 60 mmHg<br>◯ 1: 40 - 59 mmHg<br>◯ 2: 30 - 39 mmHg<br>◯ 3: < 30 mmHg
14.4	***If 'If CLTI, than...' is equal to '1' answer this question:***<br>Foot Infection (WIfI) at discharge	◯ 0: Noninfected<br>◯ 1: Mild (< 2 cm cellulitis)<br>◯ 2: Moderate (> 2 cm cellulitis / purulence)<br>◯ 3: Severe (systemic response / sepsis)
14.5	***If 'If CLTI, than...' is equal to '1' answer this question:***<br>WIfI assignment amputation image	
14.6	***If 'If CLTI, than...' is equal to '1' answer this question:***<br>WIfI assignment amputation risk at discharge	◯ 1: Very low<br>◯ 2: Low<br>◯ 3: Moderate<br>◯ 4: High
14.7	***If 'If CLTI, than...' is equal to '1' answer this question:***<br>WIfI assignment benefit revascularization image	
14.8	***If 'If CLTI, than...' is equal to '1' answer this question:***<br>WIfI assignment benefit revascularization at discharge	◯ 1: Very low<br>◯ 2: Low<br>◯ 3: Moderate<br>◯ 4: High
14.9	***If 'Fontaine classification' is equal to 'Fontaine 4 (ulceration or gangrene)' answer this question:***<br>Explanation ulcers at discharge	

15. Follow-up 6-8 weeks - Follow-up general

Number	Question	Answers
15.1	Date of visit	[ ] [ ] [ ] *(dd-mm-yyyy)*
15.1.1	***If 'Date of visit' is greater than '01-01-2000' answer this question:***<br>Visit time after intervention	
15.2	Number of visit after intervention	[ ]
15.3	Antithrombotics	☐ None<br>☐ Acetylsalicylzuur (or carbasalaatcalcium)<br>☐ P2Y12-inhibitor (e.g. clopidogrel, ticagrelor, prasugrel)<br>☐ Dipyridamol<br>☐ Vitamin K antagonist<br>☐ DOAC (e.g. rivaroxaban, dabigatran)<br>☐ Heparins
15.4	If patient uses ascal and DOAC 6wks, show DPI	
15.4.1	***If 'If patient uses ascal and DOAC 6wks, show DPI' is equal to '1' answer this question:***<br>Is the patient using rivaroxaban 2dd 2.5mg + ascal?	○ Yes<br>○ No
15.5	Specification (and indication) antithrombotics 6wks	[ ]
15.6	Major adverse cardiac events<br>*Notice shown if field's value is not equal to None: 'Please also fill in the report form "adverse events".'*	○ None<br>○ Stroke<br>○ Myocardial infarction<br>○ Death
15.7	***If 'Hospital' is equal to 'UMC Utrecht' answer this question:***<br>Did patient test on VerifyNow during visit	○ No<br>○ Yes, the clopidogrel test<br>○ Yes, the aspirin test
15.7.1	***If 'Did patient test on VerifyNow during visit' is equal to 'Yes, the clopidogrel test' answer this question:***<br>According to VerifyNow clopidogrel response	○ Good response<br>○ Bad response
15.7.2	***If 'Did patient test on VerifyNow during visit' is equal to 'Yes, the aspirin test' answer this question:***<br>According to VerifyNow aspirin response	○ Good response<br>○ Bad response

15.8	***If 'After failed attempt of revascularization, is this a case of desert foot?' is not equal to 'No, because other crural lesions have been successfully revascularized.' answer this question:***<br>If new plan after failed revascularization is a new revascularization , show attempt succesful	
15.8.1	***If 'If new plan after failed revascularization is a new revascularization , show attempt succesful' is equal to '1' answer this question:***<br>New attempt of revascularization successful?	○ Yes<br>○ No

16. Follow-up 6-8 weeks - Limb staging (including WIfI)

Number	Question	Answers
16.1	Number of ulcers visit	○ 0<br>○ 1<br>○ 2<br>○ 3<br>○ 4
16.1.1	***If 'Number of ulcers visit' is not equal to '0' answer this question:***<br>Location ulcers	☐ Calf<br>☐ Pretibial<br>☐ Ankle<br>☐ Foot<br>☐ Toe(s)<br>☐ Heel<br>☐ Unknown
16.2	Fontaine classification	○ Fontaine 1 (asymptomatic)<br>○ Fontaine 2a (pain free walking distance > 200m)<br>○ Fontaine 2b (pain free walking distance < 200m)<br>○ Fontaine 3 (rest pain)<br>○ Fontaine 4 (ulceration or gangrene)
16.3	Rutherford classification	○ 0 (asymptomatic)<br>○ 1 (mild claudation)<br>○ 2 (moderate claudication)<br>○ 3 (severe claudication)<br>○ 4 (rest pain)<br>○ 5 (minor tissue loss)<br>○ 6 (major tissue loss)

16.4	***If 'If CLTI, than...' is equal to '1' answer this question:***<br>Wound (WIfI)	◯ 0: No ulcer and no gangrene<br>◯ 1: Small ulcer and no gangrene<br>◯ 2: Deep ulcer or gangrene limited to toes<br>◯ 3: Extensive ulcer or extensive gangrene
16.5	***If 'If CLTI, than...' is equal to '1' answer this question:***<br>Ischemia (WIfI) baseline based on...	
16.6	***If 'If CLTI, than...' is equal to '1' answer this question:***<br>Ischemia (WIfI)	◯ 0: > 60 mmHg<br>◯ 1: 40 - 59 mmHg<br>◯ 2: 30 - 39 mmHg<br>◯ 3: < 30 mmHg
16.7	***If 'If CLTI, than...' is equal to '1' answer this question:***<br>Foot Infection (WIfI)	◯ 0: Noninfected<br>◯ 1: Mild (< 2 cm cellulitis)<br>◯ 2: Moderate (> 2 cm cellulitis / purulence)<br>◯ 3: Severe (systemic response / sepsis)
16.8	***If 'If CLTI, than...' is equal to '1' answer this question:***<br>WIfI assignment amputation image	
16.9	***If 'If CLTI, than...' is equal to '1' answer this question:***<br>WIfI assignment amputation risk 6wks	◯ 1: Very low<br>◯ 2: Low<br>◯ 3: Moderate<br>◯ 4: High
16.10	***If 'If CLTI, than...' is equal to '1' answer this question:***<br>WIfI assignment benefit revascularization image	
16.11	***If 'If CLTI, than...' is equal to '1' answer this question:***<br>WIfI assignment benefit revascularization 6wks	◯ 1: Very low<br>◯ 2: Low<br>◯ 3: Moderate<br>◯ 4: High
16.1.2	***If 'Number of ulcers visit' is not equal to '0' answer this question:***<br>Improvement ulcers or clinical symptoms	◯ Yes<br>◯ No, unchanged<br>◯ No, deterioration<br>◯ Unknown
16.1.3	***If 'Number of ulcers visit' is not equal to '0' answer this question:***<br>Explanation ulcers	

17. Follow-up 6-8 weeks - Radiological characteristics

Number	Question	Answers
17.1	ABI or toe pressure performed 6 wks	◯ Yes<br>◯ No
17.1.1	***If 'ABI or toe pressure performed 6 wks' is equal to 'Yes' answer this question:***<br>Blood pressure arm	
17.1.2	***If 'ABI or toe pressure performed 6 wks' is equal to 'Yes' answer this question:***<br>Arteries ankle in which ankle pressure is measured	☐ ATP<br>☐ ADP<br>☐ Unknown
17.1.2.1	***If 'Arteries ankle in which ankle pressure is measured' is equal to 'ATP' answer this question:***<br>Blood pressure ATP	
17.1.2.1.1	***If 'Blood pressure ATP' is greater or equal than '1' answer this question:***<br>ABI based on ATP pressure	
17.1.2.2	***If 'Arteries ankle in which ankle pressure is measured' is equal to 'ADP' answer this question:***<br>Blood pressure ADP	
17.1.2.2.1	***If 'Blood pressure ADP' is greater or equal than '1' answer this question:***<br>ABI based on ADP pressure	
17.1.3	***If 'ABI or toe pressure performed 6 wks' is equal to 'Yes' answer this question:***<br>Highest blood pressure in ankle 6wks	
17.1.3.1	***If 'Highest blood pressure in ankle 6wks' is greater or equal than '1' answer this question:***<br>Highest ABI 6wks	
17.1.3.2	***If 'Highest blood pressure in ankle 6wks' is smaller than '5' answer this question:***<br>Highest ABI fill in yourself	
17.1.3.3	***If 'Highest blood pressure in ankle 6wks' is smaller than '5' answer this question:***<br>Reason no ankle pressure 6wks	◯ Not compressible<br>◯ Not measured<br>◯ Other
17.1.3.3.1	***If 'Reason no ankle pressure 6wks' is equal to 'Other' answer this question:***<br>Specification reason no ankle pressure	

17.1.4	***If 'ABI or toe pressure performed 6 wks' is equal to 'Yes' answer this question:***<br>Toe pressure	
17.1.4.1	***If 'Toe pressure' is greater or equal than '1' answer this question:***<br>TBI	
17.1.4.2	***If 'Toe pressure' is greater than '-1' answer this question:***<br>Toe pressure 6wks curve	
17.1.4.3	***If 'Toe pressure' is greater than '-1' answer this question:***<br>Toe pressure acceleration time 6wks	
17.2	***If 'Toe pressure' is greater than '-1' answer this question:***<br>Ankle pressure curve (optional)	
17.3	***If 'Toe pressure' is greater than '-1' answer this question:***<br>Ankle (ATP) pressure acceleration time	
17.4	***If 'Toe pressure' is greater than '-1' answer this question:***<br>Ankle (ADP) pressure acceleration time	
17.5	Other radiology performed at 6 weeks	☐ None<br>☐ Duplex ultrasound<br>☐ MRA<br>☐ CTA<br>☐ Angiography
17.6	Was duplex ultrasound follow-up intended at 6-8 weeks?	○ No<br>○ Yes<br>○ Other
17.6.1	***If 'Was duplex ultrasound follow-up intended at 6-8 weeks?' is equal to 'Other' answer this question:***<br>Text box DUS follow-up intention	
17.5.1	***If 'Other radiology performed at 6 weeks' is equal to 'Duplex ultrasound' answer this question:***<br>Date duplex	*(dd-mm-yyyy)*
17.5.1.1	***If 'Date duplex' is greater than '01-01-2000' answer this question:***<br>Duplex time after intervention	
17.5.2	***If 'Other radiology performed at 6 weeks' is equal to 'MRA' answer this question:***<br>Date MRA	*(dd-mm-yyyy)*
17.5.2.1	***If 'Date MRA' is greater than '01-01-2000' answer this question:***<br>MRA time after intervention	

17.5.3	***If 'Other radiology performed at 6 weeks' is equal to 'CTA' answer this question:***<br>Date CTA	☐ ☐ ☐ *(dd-mm-yyyy)*
17.5.3.1	***If 'Date CTA' is greater than '01-01-2000' answer this question:***<br>CTA time after intervention	
17.5.4	***If 'Other radiology performed at 6 weeks' is equal to 'Angiography' answer this question:***<br>Date angiography	☐ ☐ ☐ *(dd-mm-yyyy)*
17.5.4.1	***If 'Date angiography' is greater than '01-01-2000' answer this question:***<br>Angiography time after intervention	
17.5.5	***If 'Other radiology performed at 6 weeks' is not equal to 'None' answer this question:***<br>Target lesion locations	
17.5.6	***If 'Other radiology performed at 6 weeks' is not equal to 'None' answer this question:***<br>Restenosis or re-occlusion in target lesions<br>*Notice shown if field's value is not equal to No: 'Please also fill in the report "adverse event".'*	☐ No<br>☐ Yes, target lesion 1<br>☐ Yes, target lesion 2<br>☐ Yes, target lesion 3<br>☐ Yes, target lesion 4<br>☐ Unknown/not visible on imaging
17.5.6.1	***If 'Restenosis or re-occlusion in target lesions' is equal to 'Yes, target lesion 1' answer this question:***<br>Target lesion 1 degree of restenosis	○ Significant restenosis (>50%)<br>○ Total re-occlusion
17.5.6.2	***If 'Restenosis or re-occlusion in target lesions' is equal to 'Yes, target lesion 2' answer this question:***<br>Target lesion 2 degree of restenosis	○ Significant restenosis (>50%)<br>○ Total re-occlusion
17.5.6.3	***If 'Restenosis or re-occlusion in target lesions' is equal to 'Yes, target lesion 3' answer this question:***<br>Target lesion 3 degree of restenosis	○ Significant restenosis (>50%)<br>○ Total re-occlusion
17.5.6.4	***If 'Restenosis or re-occlusion in target lesions' is equal to 'Yes, target lesion 4' answer this question:***<br>Target lesion 4 degree of restenosis	○ Significant restenosis (>50%)<br>○ Total re-occlusion
17.5.7	***If 'Other radiology performed at 6 weeks' is not equal to 'None' answer this question:***<br>Restenosis or re-occlusion in TAP	○ No significant restenosis (< 50%)<br>○ Yes, a significant restenosis (> 50%)<br>○ Yes, a re-occlusion<br>○ Unknown/not visible on imaging

17.5.7.1	***If 'Restenosis or re-occlusion in TAP' is not equal to 'No significant restenosis (< 50%)' answer this question:*** Location restenosis TAP	☐ AFS<br>☐ A . poplitea (P1)<br>☐ A. poplitea (P2-P3)<br>☐ ATA<br>☐ Truncus tibioperonealis<br>☐ A. peronea<br>☐ ATP<br>☐ Pedal arteries
17.5.7.2	***If 'Restenosis or re-occlusion in TAP' is not equal to 'No significant restenosis (< 50%)' answer this question:*** Specification location restenosis TAP	

18. Follow-up 6 months - Follow-up general

Number	Question	Answers
18.1	Date of visit	*(dd-mm-yyyy)*
18.1.1	***If 'Date of visit' is greater than '01-01-2000' answer this question:*** Visit time after intervention	
18.2	Number of visit after intervention	
18.3	Antithrombotics	☐ None<br>☐ Acetylsalicylzuur (or carbasalaatcalcium)<br>☐ P2Y12-inhibitor (e.g. clopidogrel, ticagrelor, prasugrel)<br>☐ Dipyridamol<br>☐ Vitamin K antagonist<br>☐ DOAC (e.g. rivaroxaban, dabigatran)<br>☐ Heparins
18.4	If patient uses ascal and DOAC 6m, show DPI	
18.4.1	***If 'If patient uses ascal and DOAC 6m, show DPI' is equal to '1' answer this question:*** Is the patient using rivaroxaban 2dd 2.5mg + ascal?	○ Yes<br>○ No
18.5	Specification (and indication) antithrombotics 6m	

18.6	Major adverse cardiac events<br>*Notice shown if field's value is not equal to None: 'Please also fill in the report form "adverse events".'*	◯ None<br>◯ Stroke<br>◯ Myocardial infarction<br>◯ Death

19. Follow-up 6 months - Limb staging (including WIfI)

Number	Question	Answers
19.1	Number of ulcers visit	◯ 0<br>◯ 1<br>◯ 2<br>◯ 3<br>◯ 4
19.1.1	***If 'Number of ulcers visit' is not equal to '0' answer this question:***<br>Location ulcers	☐ Calf<br>☐ Pretibial<br>☐ Ankle<br>☐ Foot<br>☐ Toe(s)<br>☐ Heel<br>☐ Unknown
19.2	Fontaine classification	◯ Fontaine 1 (asymptomatic)<br>◯ Fontaine 2a (pain free walking distance > 200m)<br>◯ Fontaine 2b (pain free walking distance < 200m)<br>◯ Fontaine 3 (rest pain)<br>◯ Fontaine 4 (ulceration or gangrene)
19.3	Rutherford classification	◯ 0 (asymptomatic)<br>◯ 1 (mild claudation)<br>◯ 2 (moderate claudication)<br>◯ 3 (severe claudication)<br>◯ 4 (rest pain)<br>◯ 5 (minor tissue loss)<br>◯ 6 (major tissue loss)
19.4	***If 'If CLTI, than...' is equal to '1' answer this question:***<br>Wound (WIfI)	◯ 0: No ulcer and no gangrene<br>◯ 1: Small ulcer and no gangrene<br>◯ 2: Deep ulcer or gangrene limited to toes<br>◯ 3: Extensive ulcer or extensive gangrene

19.5	***If 'If CLTI, than...' is equal to '1' answer this question:*** Ischemia (WIfI) baseline based on...	
19.6	***If 'If CLTI, than...' is equal to '1' answer this question:*** Ischemia (WIfI)	◯ 0: > 60 mmHg<br>◯ 1: 40 - 59 mmHg<br>◯ 2: 30 - 39 mmHg<br>◯ 3: < 30 mmHg
19.7	***If 'If CLTI, than...' is equal to '1' answer this question:*** Foot Infection (WIfI)	◯ 0: Noninfected<br>◯ 1: Mild (< 2 cm cellulitis)<br>◯ 2: Moderate (> 2 cm cellulitis / purulence)<br>◯ 3: Severe (systemic response / sepsis)
19.8	***If 'If CLTI, than...' is equal to '1' answer this question:*** WIfI assignment amputation image	
19.9	***If 'If CLTI, than...' is equal to '1' answer this question:*** WIfI assignment amputation risk 6m	◯ 1: Very low<br>◯ 2: Low<br>◯ 3: Moderate<br>◯ 4: High
19.10	***If 'If CLTI, than...' is equal to '1' answer this question:*** WIfI assignment benefit revascularization image	
19.11	***If 'If CLTI, than...' is equal to '1' answer this question:*** WIfI assignment benefit revascularization 6m	◯ 1: Very low<br>◯ 2: Low<br>◯ 3: Moderate<br>◯ 4: High
19.1.2	***If 'Number of ulcers visit' is not equal to '0' answer this question:*** Improvement ulcers or clinical symptoms	◯ Yes<br>◯ No, unchanged<br>◯ No, deterioration<br>◯ Unknown
19.1.3	***If 'Number of ulcers visit' is not equal to '0' answer this question:*** Explanation ulcers	

20. Follow-up 6 months - Radiological characteristics

Number	Question	Answers
20.1	ABI or toe pressure performed 6m	◯ Yes<br>◯ No

20.1.1	***If 'ABI or toe pressure performed 6m' is equal to 'Yes' answer this question:*** Blood pressure arm	
20.1.2	***If 'ABI or toe pressure performed 6m' is equal to 'Yes' answer this question:*** Arteries ankle in which ankle pressure is measured	☐ ATP<br>☐ ADP<br>☐ Unknown
20.1.2.1	***If 'Arteries ankle in which ankle pressure is measured' is equal to 'ATP' answer this question:*** Blood pressure ATP	
20.1.2.1.1	***If 'Blood pressure ATP' is greater or equal than '1' answer this question:*** ABI based on ATP pressure	
20.1.2.2	***If 'Arteries ankle in which ankle pressure is measured' is equal to 'ADP' answer this question:*** Blood pressure ADP	
20.1.2.2.1	***If 'Blood pressure ADP' is greater or equal than '1' answer this question:*** ABI based on ADP pressure	
20.1.3	***If 'ABI or toe pressure performed 6m' is equal to 'Yes' answer this question:*** Highest blood pressure in ankle 6m	
20.1.3.1	***If 'Highest blood pressure in ankle 6m' is greater or equal than '1' answer this question:*** Highest ABI 6m	
20.1.3.2	***If 'Highest blood pressure in ankle 6m' is smaller than '5' answer this question:*** Highest ABI fill in yourself	
20.1.3.3	***If 'Highest blood pressure in ankle 6m' is smaller than '5' answer this question:*** Reason no ankle pressure 6m	◯ Not compressible<br>◯ Not measured<br>◯ Other
20.1.3.3.1	***If 'Reason no ankle pressure 6m' is equal to 'Other' answer this question:*** Specification reason no ankle pressure	
20.1.4	***If 'ABI or toe pressure performed 6m' is equal to 'Yes' answer this question:*** Toe pressure	
20.1.4.1	***If 'Toe pressure' is greater or equal than '1' answer this question:*** TBI	
20.1.4.2	***If 'Toe pressure' is greater than '-1' answer this question:*** Toe pressure acceleration time 6m	

20.1.2.1.2	***If 'Blood pressure ATP' is greater than '-1' answer this question:***<br>Ankle (ATP) pressure acceleration time	
20.1.2.2.2	***If 'Blood pressure ADP' is greater than '-1' answer this question:***<br>Ankle (ADP) pressure acceleration time	
20.2	Other radiology performed at 6 months	☐ None<br>☐ Duplex ultrasound<br>☐ MRA<br>☐ CTA<br>☐ Angiography
20.2.1	***If 'Other radiology performed at 6 months' is equal to 'Duplex ultrasound' answer this question:***<br>Date duplex	*(dd-mm-yyyy)*
20.2.1.1	***If 'Date duplex' is greater than '01-01-2000' answer this question:***<br>Duplex time after intervention	
20.2.2	***If 'Other radiology performed at 6 months' is equal to 'MRA' answer this question:***<br>Date MRA	*(dd-mm-yyyy)*
20.2.2.1	***If 'Date MRA' is greater than '01-01-2000' answer this question:***<br>MRA time after intervention	
20.2.3	***If 'Other radiology performed at 6 months' is equal to 'CTA' answer this question:***<br>Date CTA	*(dd-mm-yyyy)*
20.2.3.1	***If 'Date CTA' is greater than '01-01-2000' answer this question:***<br>CTA time after intervention	
20.2.4	***If 'Other radiology performed at 6 months' is equal to 'Angiography' answer this question:***<br>Date angiography	*(dd-mm-yyyy)*
20.2.4.1	***If 'Date angiography' is greater than '01-01-2000' answer this question:***<br>Angiography time after intervention	
20.2.5	***If 'Other radiology performed at 6 months' is not equal to 'None' answer this question:***<br>Target lesion locations	

20.2.6	***If 'Other radiology performed at 6 months' is not equal to 'None' answer this question:***<br>Restenosis or re-occlusion in target lesions<br>*Notice shown if field's value is not equal to No: 'Please also fill in the report ''adverse event''.'*	☐ No<br>☐ Yes, target lesion 1<br>☐ Yes, target lesion 2<br>☐ Yes, target lesion 3<br>☐ Yes, target lesion 4<br>☐ Unknown/not visible on imaging
20.2.6.1	***If 'Restenosis or re-occlusion in target lesions' is equal to 'Yes, target lesion 1' answer this question:***<br>Target lesion 1 degree of restenosis	○ Significant restenosis (>50%)<br>○ Total re-occlusion
20.2.6.2	***If 'Restenosis or re-occlusion in target lesions' is equal to 'Yes, target lesion 2' answer this question:***<br>Target lesion 2 degree of restenosis	○ Significant restenosis (>50%)<br>○ Total re-occlusion
20.2.6.3	***If 'Restenosis or re-occlusion in target lesions' is equal to 'Yes, target lesion 3' answer this question:***<br>Target lesion 3 degree of restenosis	○ Significant restenosis (>50%)<br>○ Total re-occlusion
20.2.6.4	***If 'Restenosis or re-occlusion in target lesions' is equal to 'Yes, target lesion 4' answer this question:***<br>Target lesion 4 degree of restenosis	○ Significant restenosis (>50%)<br>○ Total re-occlusion
20.2.7	***If 'Other radiology performed at 6 months' is not equal to 'None' answer this question:***<br>Restenosis or re-occlusion in TAP	○ No significant restenosis (< 50%)<br>○ Yes, a significant restenosis (> 50%)<br>○ Yes, a re-occlusion<br>○ Unknown/not visible on imaging
20.2.7.1	***If 'Restenosis or re-occlusion in TAP' is not equal to 'No significant restenosis (< 50%)' answer this question:***<br>Location restenosis TAP	☐ AFS<br>☐ A . poplitea (P1)<br>☐ A. poplitea (P2-P3)<br>☐ ATA<br>☐ Truncus tibioperonealis<br>☐ A. peronea<br>☐ ATP<br>☐ Pedal arteries
20.2.7.2	***If 'Restenosis or re-occlusion in TAP' is not equal to 'No significant restenosis (< 50%)' answer this question:***<br>Specification location restenosis TAP	

21. Follow-up 1 year - Follow-up general

Number	Question	Answers
21.1	Date of visit	___ ___ ___ *(dd-mm-yyyy)*

21.1.1	***If 'Date of visit' is greater than '01-01-2000' answer this question:***<br>Visit time after intervention	
21.2	Number of visit after intervention	
21.3	Antithrombotics	☐ None<br>☐ Acetylsalicylzuur (or carbasalaatcalcium)<br>☐ P2Y12-inhibitor (e.g. clopidogrel, ticagrelor, prasugrel)<br>☐ Dipyridamol<br>☐ Vitamin K antagonist<br>☐ DOAC (e.g. rivaroxaban, dabigatran)<br>☐ Heparins
21.4	If patient uses ascal and DOAC 1y, show DPI	
21.4.1	***If 'If patient uses ascal and DOAC 1y, show DPI' is equal to '1' answer this question:***<br>Is the patient using rivaroxaban 2dd 2.5mg + ascal?	◯ Yes<br>◯ No
21.5	Specification (and indication) antithrombotics 1y	
21.6	Major adverse cardiac events<br>*Notice shown if field's value is not equal to None: 'Please also fill in the report form "adverse events".'*	◯ None<br>◯ Stroke<br>◯ Myocardial infarction<br>◯ Death
21.7	***If 'Hospital' is equal to 'UMC Utrecht' answer this question:***<br>Did patient test on VerifyNow during visit	◯ No<br>◯ Yes, the clopidogrel test<br>◯ Yes, the aspirin test
21.7.1	***If 'Did patient test on VerifyNow during visit' is equal to 'Yes, the clopidogrel test' answer this question:***<br>According to VerifyNow clopidogrel response	◯ Good response<br>◯ Bad response
21.7.2	***If 'Did patient test on VerifyNow during visit' is equal to 'Yes, the aspirin test' answer this question:***<br>According to VerifyNow aspirin response	◯ Good response<br>◯ Bad response

22. Follow-up 1 year - Limb staging

Number	Question	Answers

22.1	Number of wounds visit	◯ 0<br>◯ 1<br>◯ 2<br>◯ 3<br>◯ 4
22.1.1	***If 'Number of wounds visit' is not equal to '0' answer this question:***<br>Location wounds	☐ Calf<br>☐ Pretibial<br>☐ Ankle<br>☐ Foot<br>☐ Toe(s)<br>☐ Heel<br>☐ Unknown
22.2	Fontaine classification	◯ Fontaine 1 (asymptomatic)<br>◯ Fontaine 2a (pain free walking distance > 200m)<br>◯ Fontaine 2b (pain free walking distance < 200m)<br>◯ Fontaine 3 (rest pain)<br>◯ Fontaine 4 (ulceration or gangrene)
22.3	Rutherford classification	◯ 0 (asymptomatic)<br>◯ 1 (mild claudation)<br>◯ 2 (moderate claudication)<br>◯ 3 (severe claudication)<br>◯ 4 (rest pain)<br>◯ 5 (minor tissue loss)<br>◯ 6 (major tissue loss)
22.4	***If 'If CLTI, than...' is equal to '1' answer this question:***<br>Wound (WIfI)	◯ 0: No ulcer and no gangrene<br>◯ 1: Small ulcer and no gangrene<br>◯ 2: Deep ulcer or gangrene limited to toes<br>◯ 3: Extensive ulcer or extensive gangrene
22.5	***If 'If CLTI, than...' is equal to '1' answer this question:***<br>Ischemia (WIfI) baseline based on...	
22.6	***If 'If CLTI, than...' is equal to '1' answer this question:***<br>Ischemia (WIfI)	◯ 0: > 60 mmHg<br>◯ 1: 40 - 59 mmHg<br>◯ 2: 30 - 39 mmHg<br>◯ 3: < 30 mmHg

22.7	***If 'If CLTI, than...' is equal to '1' answer this question:***<br>Foot Infection (WIfI)	◯ 0: Noninfected<br>◯ 1: Mild (< 2 cm cellulitis)<br>◯ 2: Moderate (> 2 cm cellulitis / purulence)<br>◯ 3: Severe (systemic response / sepsis)
22.8	***If 'If CLTI, than...' is equal to '1' answer this question:***<br>WIfI assignment amputation image	
22.9	***If 'If CLTI, than...' is equal to '1' answer this question:***<br>WIfI assignment amputation risk 1y	◯ 1: Very low<br>◯ 2: Low<br>◯ 3: Moderate<br>◯ 4: High
22.10	***If 'If CLTI, than...' is equal to '1' answer this question:***<br>WIfI assignment benefit revascularization image	
22.11	***If 'If CLTI, than...' is equal to '1' answer this question:***<br>WIfI assignment benefit revascularization 1y	◯ 1: Very low<br>◯ 2: Low<br>◯ 3: Moderate<br>◯ 4: High
22.1.2	***If 'Number of wounds visit' is not equal to '0' answer this question:***<br>Improvement ulcers or clinical symptoms	◯ Yes<br>◯ No, unchanged<br>◯ No, deterioration<br>◯ Unknown
22.1.3	***If 'Number of wounds visit' is not equal to '0' answer this question:***<br>Explanation ulcers	

23. Follow-up 1 year - Radiological characteristics

Number	Question	Answers
23.1	ABI performed 1 year	◯ Yes<br>◯ No
23.1.1	***If 'ABI performed 1 year' is equal to 'Yes' answer this question:***<br>Blood pressure arm	
23.1.2	***If 'ABI performed 1 year' is equal to 'Yes' answer this question:***<br>Arteries ankle in which ankle pressure is measured	☐ ATP<br>☐ ADP<br>☐ Unknown

23.1.2.1	***If 'Arteries ankle in which ankle pressure is measured' is equal to 'ATP' answer this question:***<br>Blood pressure ATP	
23.1.2.1.1	***If 'Blood pressure ATP' is greater or equal than '1' answer this question:***<br>ABI based on ATP pressure	
23.1.2.2	***If 'Arteries ankle in which ankle pressure is measured' is equal to 'ADP' answer this question:***<br>Blood pressure ADP	
23.1.2.2.1	***If 'Blood pressure ADP' is greater or equal than '1' answer this question:***<br>ABI based on ADP pressure	
23.1.3	***If 'ABI performed 1 year' is equal to 'Yes' answer this question:***<br>Highest blood pressure in ankle 1 year	
23.1.3.1	***If 'Highest blood pressure in ankle 1 year' is greater or equal than '1' answer this question:***<br>Highest ABI 1 year	
23.1.3.2	***If 'Highest blood pressure in ankle 1 year' is smaller than '5' answer this question:***<br>Highest ABI fill in yourself	
23.1.3.3	***If 'Highest blood pressure in ankle 1 year' is smaller than '5' answer this question:***<br>Reason no ankle pressure	○ Not compressible<br>○ Not measured<br>○ Other
23.1.3.3.1	***If 'Reason no ankle pressure' is equal to 'Other' answer this question:***<br>Specification reason no ankle pressure	
23.1.4	***If 'ABI performed 1 year' is equal to 'Yes' answer this question:***<br>Toe pressure	
23.1.4.1	***If 'Toe pressure' is greater or equal than '1' answer this question:***<br>TBI	
23.1.4.2	***If 'Toe pressure' is greater than '-1' answer this question:***<br>Toe pressure 1y curve	
23.1.4.3	***If 'Toe pressure' is greater than '-1' answer this question:***<br>Toe pressure acceleration time 1y	
23.2	***If 'Toe pressure' is greater than '-1' answer this question:***<br>Ankle pressure curve (optional)	
23.3	***If 'Toe pressure' is greater than '-1' answer this question:***<br>Ankle (ATP) pressure acceleration time	

23.4	***If 'Toe pressure' is greater than '-1' answer this question:***<br>Ankle (ADP) pressure acceleration time	
23.5	Other imaging performed 1 year	☐ None<br>☐ Duplex ultrasound<br>☐ MRA<br>☐ CTA<br>☐ Angiography
23.6	Was duplex ultrasound follow-up intended at about 1 year?	○ No<br>○ Yes<br>○ Other
23.6.1	***If 'Was duplex ultrasound follow-up intended at about 1 year?' is equal to 'Other' answer this question:***<br>Text box DUS follow-up intention	
23.5.1	***If 'Other imaging performed 1 year' is equal to 'Duplex ultrasound' answer this question:***<br>Date duplex	*(dd-mm-yyyy)*
23.5.1.1	***If 'Date duplex' is greater than '01-01-2000' answer this question:***<br>Duplex time after intervention	
23.5.2	***If 'Other imaging performed 1 year' is equal to 'MRA' answer this question:***<br>Date MRA	*(dd-mm-yyyy)*
23.5.2.1	***If 'Date MRA' is greater than '01-01-2000' answer this question:***<br>MRA time after intervention	
23.5.3	***If 'Other imaging performed 1 year' is equal to 'CTA' answer this question:***<br>Date CTA	*(dd-mm-yyyy)*
23.5.3.1	***If 'Date CTA' is greater than '01-01-2000' answer this question:***<br>CTA time after intervention	
23.5.4	***If 'Other imaging performed 1 year' is equal to 'Angiography' answer this question:***<br>Date angiography	*(dd-mm-yyyy)*
23.5.4.1	***If 'Date angiography' is greater than '01-01-2000' answer this question:***<br>Angiography time after intervention	
23.5.5	***If 'Other imaging performed 1 year' is not equal to 'None' answer this question:***<br>Target lesion locations	

23.5.6	***If 'Other imaging performed 1 year' is not equal to 'None' answer this question:***<br>Restenosis or re-occlusion in target lesions<br>*Notice shown if field's value is not equal to No: 'Please also fill in the report ''adverse event''.'*	☐ No<br>☐ Yes, target lesion 1<br>☐ Yes, target lesion 2<br>☐ Yes, target lesion 3<br>☐ Yes, target lesion 4<br>☐ Unknown/not visible on imaging
23.5.6.1	***If 'Restenosis or re-occlusion in target lesions' is equal to 'Yes, target lesion 1' answer this question:***<br>Target lesion 1 degree of restenosis	○ Significant restenosis (>50%)<br>○ Total re-occlusion
23.5.6.2	***If 'Restenosis or re-occlusion in target lesions' is equal to 'Yes, target lesion 2' answer this question:***<br>Target lesion 2 degree of restenosis	○ Significant restenosis (>50%)<br>○ Total re-occlusion
23.5.6.3	***If 'Restenosis or re-occlusion in target lesions' is equal to 'Yes, target lesion 3' answer this question:***<br>Target lesion 3 degree of restenosis	○ Significant restenosis (>50%)<br>○ Total re-occlusion
23.5.6.4	***If 'Restenosis or re-occlusion in target lesions' is equal to 'Yes, target lesion 4' answer this question:***<br>Target lesion 4 degree of restenosis	○ Significant restenosis (>50%)<br>○ Total re-occlusion
23.5.7	***If 'Other imaging performed 1 year' is not equal to 'None' answer this question:***<br>Restenosis or re-occlusion in TAP	○ No significant restenosis (< 50%)<br>○ Yes, a significant restenosis (> 50%)<br>○ Yes, a re-occlusion<br>○ Unknown/not visible on imaging
23.5.7.1	***If 'Restenosis or re-occlusion in TAP' is not equal to 'No significant restenosis (< 50%)' answer this question:***<br>Location restenosis TAP	☐ AFS<br>☐ A . poplitea (P1)<br>☐ A. poplitea (P2-P3)<br>☐ ATA<br>☐ Truncus tibioperonealis<br>☐ A. peronea<br>☐ ATP<br>☐ Pedal arteries
23.5.7.2	***If 'Restenosis or re-occlusion in TAP' is not equal to 'No significant restenosis (< 50%)' answer this question:***<br>Specification location restenosis TAP	

24. Follow-up 2 year - Follow-up general

Number	Question	Answers
24.1	Date of visit	__ __ __ *(dd-mm-yyyy)*

24.1.1	***If 'Date of visit' is greater than '01-01-2000' answer this question:***<br>Visit time after intervention	
24.2	Number of visit after intervention	
24.3	Antithrombotics	☐ None<br>☐ Acetylsalicylzuur (or carbasalaatcalcium)<br>☐ P2Y12-inhibitor (e.g. clopidogrel, ticagrelor, prasugrel)<br>☐ Dipyridamol<br>☐ Vitamin K antagonist<br>☐ DOAC (e.g. rivaroxaban, dabigatran)<br>☐ Heparins
24.4	If patient uses ascal and DOAC 2y, show DPI	
24.4.1	***If 'If patient uses ascal and DOAC 2y, show DPI' is equal to '1' answer this question:***<br>Is the patient using rivaroxaban 2dd 2.5mg + ascal?	○ Yes<br>○ No
24.5	Specification (and indication) antithrombotics 2y	
24.6	Major adverse cardiac events<br>*Notice shown if field's value is not equal to None: 'Please also fill in the report form "adverse events".'*	○ None<br>○ Stroke<br>○ Myocardial infarction<br>○ Death

25. Follow-up 2 year - Limb staging

Number	Question	Answers
25.1	Number of wounds visit	○ 0<br>○ 1<br>○ 2<br>○ 3<br>○ 4

25.1.1	***If 'Number of wounds visit' is not equal to '0' answer this question:***<br>Location wounds	☐ Calf<br>☐ Pretibial<br>☐ Ankle<br>☐ Foot<br>☐ Toe(s)<br>☐ Heel<br>☐ Unknown
25.2	Fontaine classification	○ Fontaine 1 (asymptomatic)<br>○ Fontaine 2a (pain free walking distance > 200m)<br>○ Fontaine 2b (pain free walking distance < 200m)<br>○ Fontaine 3 (rest pain)<br>○ Fontaine 4 (ulceration or gangrene)
25.3	Rutherford classification	○ 0 (asymptomatic)<br>○ 1 (mild claudation)<br>○ 2 (moderate claudication)<br>○ 3 (severe claudication)<br>○ 4 (rest pain)<br>○ 5 (minor tissue loss)<br>○ 6 (major tissue loss)
25.4	Wound (WIfI)	○ 0: No ulcer and no gangrene<br>○ 1: Small ulcer and no gangrene<br>○ 2: Deep ulcer or gangrene limited to toes<br>○ 3: Extensive ulcer or extensive gangrene
25.5	Ischemia (WIfI) baseline based on...	
25.6	Ischemia (WIfI)	○ 0: > 60 mmHg<br>○ 1: 40 - 59 mmHg<br>○ 2: 30 - 39 mmHg<br>○ 3: < 30 mmHg
25.7	Foot Infection (WIfI)	○ 0: Noninfected<br>○ 1: Mild (< 2 cm cellulitis)<br>○ 2: Moderate (> 2 cm cellulitis / purulence)<br>○ 3: Severe (systemic response / sepsis)
25.8	WIfI assignment amputation image	

25.9	WIfI assignment amputation risk 2y	◯ 1: Very low<br>◯ 2: Low<br>◯ 3: Moderate<br>◯ 4: High
25.10	WIfI assignment benefit revascularization image	
25.11	WIfI assignment benefit revascularization 2y	◯ 1: Very low<br>◯ 2: Low<br>◯ 3: Moderate<br>◯ 4: High
25.1.2	***If 'Number of wounds visit' is not equal to '0' answer this question:***<br>Explanation ulcers	

26. Follow-up 2 year - Radiological characteristics

Number	Question	Answers
26.1	ABI performed 2 years	◯ Yes<br>◯ No
26.1.1	***If 'ABI performed 2 years' is equal to 'Yes' answer this question:***<br>Blood pressure arm	
26.1.2	***If 'ABI performed 2 years' is equal to 'Yes' answer this question:***<br>Arteries ankle in which ankle pressure is measured	☐ ATP<br>☐ ADP<br>☐ Unknown
26.1.2.1	***If 'Arteries ankle in which ankle pressure is measured' is equal to 'ATP' answer this question:***<br>Blood pressure ATP	
26.1.2.1.1	***If 'Blood pressure ATP' is greater or equal than '1' answer this question:***<br>ABI based on ATP pressure	
26.1.2.2	***If 'Arteries ankle in which ankle pressure is measured' is equal to 'ADP' answer this question:***<br>Blood pressure ADP	
26.1.2.2.1	***If 'Blood pressure ADP' is greater or equal than '1' answer this question:***<br>ABI based on ADP pressure	

26.1.3	***If 'ABI performed 2 years' is equal to 'Yes' answer this question:***<br>Highest blood pressure in ankle 2 years	
26.1.3.1	***If 'Highest blood pressure in ankle 2 years' is greater or equal than '1' answer this question:***<br>Highest ABI 1 year	
26.1.3.2	***If 'Highest blood pressure in ankle 2 years' is smaller than '5' answer this question:***<br>Highest ABI fill in yourself	
26.1.3.3	***If 'Highest blood pressure in ankle 2 years' is smaller than '5' answer this question:***<br>Reason no ankle pressure	○ Not compressible<br>○ Not measured<br>○ Other
26.1.3.3.1	***If 'Reason no ankle pressure' is equal to 'Other' answer this question:***<br>Specification reason no ankle pressure	
26.1.4	***If 'ABI performed 2 years' is equal to 'Yes' answer this question:***<br>Toe pressure	
26.1.4.1	***If 'Toe pressure' is greater or equal than '1' answer this question:***<br>TBI	
26.2	Other imaging performed 2 years	☐ None<br>☐ Duplex ultrasound<br>☐ MRA<br>☐ CTA<br>☐ Angiography
26.3	Was duplex ultrasound follow-up intended at about 2 years?	○ No<br>○ Yes<br>○ Other
26.3.1	***If 'Was duplex ultrasound follow-up intended at about 2 years?' is equal to 'Other' answer this question:***<br>Text box DUS follow-up intention	
26.2.1	***If 'Other imaging performed 2 years' is equal to 'Duplex ultrasound' answer this question:***<br>Date duplex	*(dd-mm-yyyy)*
26.2.1.1	***If 'Date duplex' is greater than '01-01-2000' answer this question:***<br>Duplex time after intervention	

26.2.2	***If 'Other imaging performed 2 years' is equal to 'MRA' answer this question:***<br>Date MRA	☐ ☐ ☐ *(dd-mm-yyyy)*
26.2.2.1	***If 'Date MRA' is greater than '01-01-2000' answer this question:***<br>MRA time after intervention	
26.2.3	***If 'Other imaging performed 2 years' is equal to 'CTA' answer this question:***<br>Date CTA	☐ ☐ ☐ *(dd-mm-yyyy)*
26.2.3.1	***If 'Date CTA' is greater than '01-01-2000' answer this question:***<br>CTA time after intervention	
26.2.4	***If 'Other imaging performed 2 years' is equal to 'Angiography' answer this question:***<br>Date angiography	☐ ☐ ☐ *(dd-mm-yyyy)*
26.2.4.1	***If 'Date angiography' is greater than '01-01-2000' answer this question:***<br>Angiography time after intervention	
26.2.5	***If 'Other imaging performed 2 years' is not equal to 'None' answer this question:***<br>Target lesion locations	
26.2.6	***If 'Other imaging performed 2 years' is not equal to 'None' answer this question:***<br>Restenosis or re-occlusion in target lesions<br>*Notice shown if field's value is not equal to No: 'Please also fill in the report ''adverse event''.'*	☐ No<br>☐ Yes, target lesion 1<br>☐ Yes, target lesion 2<br>☐ Yes, target lesion 3<br>☐ Yes, target lesion 4<br>☐ Unknown/not visible on imaging
26.2.6.1	***If 'Restenosis or re-occlusion in target lesions' is equal to 'Yes, target lesion 1' answer this question:***<br>Target lesion 1 degree of restenosis	○ Significant restenosis (>50%)<br>○ Total re-occlusion
26.2.6.2	***If 'Restenosis or re-occlusion in target lesions' is equal to 'Yes, target lesion 2' answer this question:***<br>Target lesion 2 degree of restenosis	○ Significant restenosis (>50%)<br>○ Total re-occlusion
26.2.6.3	***If 'Restenosis or re-occlusion in target lesions' is equal to 'Yes, target lesion 3' answer this question:***<br>Target lesion 3 degree of restenosis	○ Significant restenosis (>50%)<br>○ Total re-occlusion
26.2.6.4	***If 'Restenosis or re-occlusion in target lesions' is equal to 'Yes, target lesion 4' answer this question:***<br>Target lesion 4 degree of restenosis	○ Significant restenosis (>50%)<br>○ Total re-occlusion
26.2.7	***If 'Other imaging performed 2 years' is not equal to 'None' answer this question:***<br>Restenosis or re-occlusion in TAP	○ No significant restenosis (< 50%)<br>○ Yes, a significant restenosis (> 50%)<br>○ Yes, a re-occlusion<br>○ Unknown/not visible on imaging

Number	Question	Answers
26.2.7.1	***If 'Restenosis or re-occlusion in TAP' is not equal to 'No significant restenosis (< 50%)' answer this question:***<br>Location restenosis TAP	☐ AFS<br>☐ A . poplitea (P1)<br>☐ A. poplitea (P2-P3)<br>☐ ATA<br>☐ Truncus tibioperonealis<br>☐ A. peronea<br>☐ ATP<br>☐ Pedal arteries
26.2.7.2	***If 'Restenosis or re-occlusion in TAP' is not equal to 'No significant restenosis (< 50%)' answer this question:***<br>Specification location restenosis TAP	

27. Date last visit - Date last visit

Number	Question	Answers
27.1	Date last visit or death	*(dd-mm-yyyy)*
27.1.1	***If 'Date last visit or death' is greater than '01-01-2000' answer this question:***<br>Time until last visit or death	
27.2	Did the patient decease?	○ Yes<br>○ No
27.3	Date last imaging	*(dd-mm-yyyy)*
27.3.1	***If 'Date last imaging' is greater than '01-01-2000' answer this question:***<br>Time until last imaging	
27.4	***If 'Number of ulcers baseline' is not equal to '0' answer this question:***<br>Date wound healing	*(dd-mm-yyyy)*
27.4.1	***If 'Date wound healing' is greater than '01-01-2000' answer this question:***<br>Time until wound healing	
27.5	***If 'Number of ulcers baseline' is not equal to '0' answer this question:***<br>Did the wound heal?	○ Yes<br>○ No
27.6	Date last visit, death or major amputation	*(dd-mm-yyyy)*
27.6.1	***If 'Date last visit, death or major amputation' is greater than '01-01-2000' answer this question:***<br>Time until last visit or major amputation	

27.7	Did the patient undergo a major amputation?	◯ Yes<br>◯ No
27.8	Did the patient undergo a major amputation or deceased?	◯ Yes<br>◯ No
